# *ABCA4* Variant c.5714+5G>A in *Trans* With Null Alleles Results in Primary RPE Damage

**DOI:** 10.1167/iovs.64.12.33

**Published:** 2023-09-20

**Authors:** Jana Sajovic, Andrej Meglič, Zelia Corradi, Mubeen Khan, Aleš Maver, Martina Jarc Vidmar, Marko Hawlina, Frans P. M. Cremers, Ana Fakin

**Affiliations:** 1 Eye Hospital, University Medical Centre Ljubljana, Ljubljana, Slovenia; 2 Faculty of Medicine, University of Ljubljana, Ljubljana, Slovenia; 3 Department of Human Genetics, Radboud University Medical Center, Nijmegen, the Netherlands; 4 Donders Institute for Brain, Cognition and Behaviour, Radboud University, Nijmegen, the Netherlands; 5 Max Planck Institute for Psycholinguistics, Nijmegen, the Netherlands; 6 Clinical Institute of Genomic Medicine, University Medical Centre Ljubljana, Ljubljana, Slovenia

**Keywords:** STGD1, c.5714+5G>A, splicing, PERG, autofluorescence

## Abstract

**Purpose:**

To determine the disease pathogenesis associated with the frequent *ABCA4* variant c.5714+5G>A (p.[=,Glu1863Leufs*33]).

**Methods:**

Patient-derived photoreceptor precursor cells were generated to analyze the effect of c.5714+5G>A on splicing and perform a quantitative analysis of c.5714+5G>A products. Patients with c.5714+5G>A in *trans* with a null allele (i.e., c.5714+5G>A patients; *n* = 7) were compared with patients with two null alleles (i.e., double null patients; *n* = 11); with a special attention to the degree of RPE atrophy (area of definitely decreased autofluorescence and the degree of photoreceptor impairment (outer nuclear layer thickness and pattern electroretinography amplitude).

**Results:**

RT-PCR of mRNA from patient-derived photoreceptor precursor cells showed exon 40 and exon 39/40 deletion products, as well as the normal transcript. Quantification of products showed 52.4% normal and 47.6% mutant *ABCA4* mRNA. Clinically, c.5714+5G>A patients displayed significantly better structural and functional preservation of photoreceptors (thicker outer nuclear layer, presence of tubulations, higher pattern electroretinography amplitude) than double null patients with similar degrees of RPE loss, whereas double null patients exhibited signs of extensive photoreceptor ,damage even in the areas with preserved RPE.

**Conclusions:**

The prototypical STGD1 sequence of events of primary RPE and secondary photoreceptor damage is congruous with c.5714+5G>A, but not the double null genotype, which implies different and genotype-dependent disease mechanisms. We hypothesize that the relative photoreceptor sparing in c.5714+5G>A patients results from the remaining function of the ABCA4 transporter originating from the normally spliced product, possibly by decreasing the direct bisretinoid toxicity on photoreceptor membranes.

Stargardt disease (STGD1), also known as *ABCA4* retinopathy, is the most frequent retinal dystrophy caused by a single gene, affecting approximately 1 in 8000 to 10,000 people.^1^ The ABCA4 protein, encoded by the *ABCA4* gene, is situated in rod and cone photoreceptor outer segment disks and lamellae, respectively^2^^–^^5^ and functions as a transmembrane transporter of molecules involved in the visual transduction.^6^^,^^7^ Despite this progress and improvements in our understanding of STGD1, the pathogenesis is still poorly understood, which is precluding the development of treatment strategies.

STGD1 is known for a very heterogeneous phenotypic appearance,^8^^–^^10^ which is reflected in numerous clinical classifications, such as categorization according to fundus appearance (Fishman classification),^11^ electroretinography (ERG),^12^ presence or absence of foveal atrophy,^8^^,^^9^^,^^13^^–^^18^ and age at onset.^15^

With the widespread availability of genetic diagnosis, attempts are being made to connect the clinical variability with the nature of the causative alleles.^8^^,^^9^^,^^13^^,^^14^^,^^19^^–^^22^ Patients with two null alleles have been shown to have the most severe disease, that is, childhood onset cone–rod dystrophy.^13^

Characterization of other, non-null alleles has been more challenging^8^^,^^23^ because there are >2000 disease-associated variants (www.lovd.nl/ABCA4, accessed May 8, 2023) that, owing to the recessive nature of disease, combine in thousands of different pairings. Characterisation of *ABCA4* variants is further complicated owing to limitations in performing functional assays.

Retinal tissue of living patients is not readily available and *ABCA4* gene expression in accessible somatic cells is very low^24^^–^^26^; therefore, other approaches, such as in vitro assays or patient-derived photoreceptor precursor cells (PPCs) need to be used to study the effect of different variants.^27^^–^^29^

The noncanonical splice site variant c.5714+5G>A (p.[=,Glu1863Leufs*33]), located in the 5′ splice region of intron 40,^30^ is the sixth most frequent variant in patients with STGD1,^31^ identified in 1% to 16% of cases^9^^,^^32^^–^^35^ and recurrent in Newfoundland cases owing to a major founder effect.^36^ Despite that, the effect of c.5714+5G>A variant on structural and functional retinal changes has not yet been systematically addressed.

In the Slovenian registry of patients with *ABCA4* retinopathy, c.5714+5G>A is the fourth most frequent variant, present in 12% of patients, which provides an opportunity to perform a detailed phenotypic specification. Moreover, an ex vivo assay using PPC has been developed at the Radboudumc Stem Cell Technology Center in the Netherlands to study the variant's effect in conditions most closely resembling in vivo situation. Combining the two approaches, this study provides insights into disease pathogenesis associated with one of the most frequent *ABCA4* variants.

## Methods

### Ethics and Declarations

This study was conducted according to the tenets of the Declaration of Helsinki. The procedures for obtaining human blood samples for ex vivo part were approved by the local Ethical Committee in Nijmegen (protocol ID number: 2018–4516). The clinical part was reviewed and approved by the National Medical Ethics Committee of the Republic of Slovenia (protocol ID number: 0120-50/2021/3). Written informed consent was obtained from participants before their enrollment.

### Ex Vivo Analysis

#### Epstein–Barr Virus (EBV) Transformation of Human Blood

Blood was collected from one control individual and an individual with STGD1 carrying the *ABCA4* variants c.5714+5G>A; p.[=,Glu1863Leufs*33] and c.4539+1G>T; p.(?). Stable lymphoblastoid cell lines were obtained from the peripheral blood of the control and STGD1 individual by EBV transformation following the previously described protocol.^37^ Briefly, white blood cells were isolated from blood samples and resuspended in EBV supernatant. After 2 hours of incubation, cells were pelleted, resuspended with cyclosporine solution, and placed to grow in 24-well plates in a 37°C and 7.5% CO_2_ incubator. After 2 weeks, the cultures were assessed with a microscope.

#### Generation of Induced Pluripotent Stem Cells and Differentiation Into Photoreceptor Precursor Cells

EBV immortalized lymphoblasts were cultured in mononuclear cell medium for 5 days in a T75 flask. Subsequently, reprogramming into induced pluripotent stem cells was performed following a previously described method. In short, four lentiviral vectors containing the pluripotency genes OCT3/4, NANOG, *KLF4*, and c-MYC were used for the transduction of EBV lymphoblasts. The cells were then cultured on growth factor-reduced Matrigel (Corning Life Sciences, Tewksbury, MA, USA) in Essential 8 Flex medium (Thermo Fisher Scientific, Waltham, MA, USA) and passaged as clumps at a ratio of 1:5 to 1:10 every 5 to 6 days by digestion with 0.5 mM EDTA. Cells were maintained at 37°C and 5% CO_2_. At passage 10, immunocytochemistry and quantitative RT-PCR were performed to characterize induced pluripotent stem cells and assess pluripotency markers as described in the protocol (see Supplementary Figs. S1, S2, and S3).^38^

For differentiation, the Flamier et al.^39^ protocol was followed. DMEM/F12 medium was supplemented with non-essential amino acids (NEAA; Sigma-Aldrich, St. Louis, MO, USA), N2 supplements (Thermo Fisher Scientific), B27 supplements (Thermo Fisher Scientific), heparin (Sigma-Aldrich), insulin-like growth factor-1 (Sigma-Aldrich), recombinant basic FGF (STEMCELL Technologies, Vancouver, Canada), and human recombinant COCO (R&D Systems, Inc., Minneapolis, MN, USA). The cells were characterized after 1 month of differentiation, during which the medium was changed daily.

#### Induced Pluripotent Stem Cells and Photoreceptor Precursor Cells Characterization

The characterization of the patient and control lines was performed by comparing expression of differentiation markers between induced pluripotent stem cells and PPCs by quantitative PCR (see Supplementary Fig. S4). RNA was isolated from collected cells using the NucleoSpin RNA Clean-up Kit (Macherey-Nagel, Duren, Germany) and cDNA was synthesized with the iScript cDNA synthesis kit (Bio-Rad Laboratories, Hercules, CA, USA) starting from 1 µg of RNA. For both experiments the manufacturer's protocol were followed.

Quantitative PCR was preformed using GoTaq Real-Time quantitative PCR Master Kit (Promega, Madison, WI, USA). The primers for the selected differentiation markers and the housekeeping gene *GUSB* are reported in Supplementary Table S1. Each reaction was set up in triplicate and performed in technical duplicate using the 7900HT fast real-time PCR system. The 2^−(ΔΔCt)^ method was used to assess the relative expression of the markers, values are reported as the average with SD of the technical duplicates.

#### *ABCA4* Transcript Analysis

For *ABCA4* transcript analysis, PPCs derived from the healthy individual and the STGD1 proband were harvested after 30 days of differentiation. Cells were treated with cycloheximide (CHX; Sigma-Aldrich) 24 hours before collection to suppress nonsense-mediated decay. RNA isolation and cDNA synthesis were performed as described above.

RT-PCR analysis was performed using primers located in exon 38 (forward) and exon 44 (reverse) of *ABCA4*. The following conditions were used to perform RT-PCR: 94°C for 2 minutes, 35 cycles of 30 seconds at 94°C, 30 seconds at 58°C, 90 seconds at 72°C, and a final elongation of 2 minutes at 72°C. Actin Beta (*ACTB*) was used as house-keeping gene.

RT-PCR products were resolved on 2% agarose gel, purified using the Nucleospin Gel and PCR Clean-up Kit (Macherey-Nagel, Duren, Germany) and analyzed by Sanger sequencing. Fiji software^40^ was used to perform semi-quantification of the ratio between different transcripts. Semiquantification was done in a technical duplicate (see Supplementary Table S2) on the most representative biological.

### Clinical Analysis

#### Patients

The study included 18 patients with a clinical and molecular diagnosis of STGD1, recruited from the STGD1 cohort overseen at the Eye Hospital, University Medical Centre Ljubljana based on their genotype. The main study group (c.5714+5G>A patients; *n* = 7; 4 males), were patients with the splicing variant c.5714+5G>A in *trans* with a null variant, which allowed us to study this variant in isolation. The control group were patients with two null variants (double null patients; *n* = 11; 3 males). For the purpose of this study, a variant was considered to be null if it was either a stop variant, a frame-shifting variant resulting in a stop codon, or splicing and missense variants previously shown to behave as null.^13^ The specific patients' variants are listed in Supplementary Table S3. In a group of patients with c.5714+5G>A allele, there were five unrelated patients and one sibling pair, whereas the group of double null patients consisted of seven unrelated patients and two sibling pairs.

#### Clinical Evaluation

**Figure 1.**
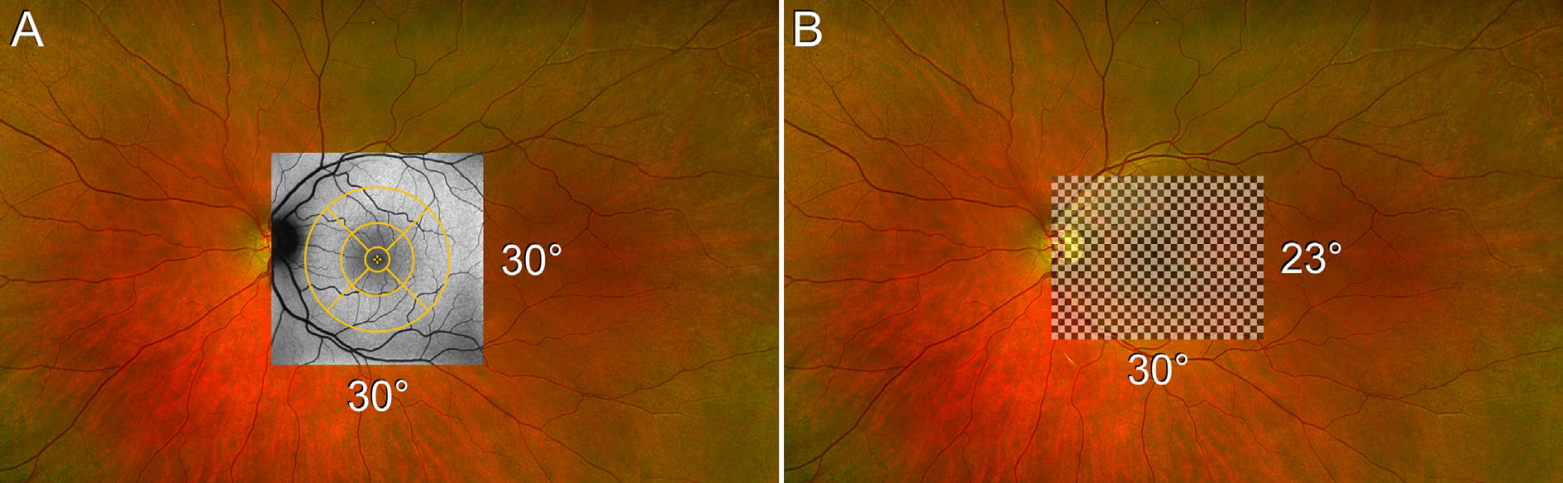
Analyzed retinal area. Schematic representation of the area of the retina imaged using 30° FAF imaging (for assessing RPE damage) and SD-OCT (for assessing photoreceptor damage) (**A**) and stimulated when performing PERG (for assessing photoreceptor function) (**B**).

Clinical evaluation included age at onset (age when patients noted loss of visual acuity), disease duration (difference between the age at examination and age of onset), best-corrected visual acuity (BCVA), ERG (Diagnosys LLC, Littleton, MA, USA; recorded according to the standards of the International Society of Clinical Electrophysiology of Vision^41^^,^^42^; fundus autofluorescence (FAF) and SD-OCT (Heidelberg Engineering, Heidelberg, Germany). A similar retinal area of approximately 30° × 30° was analyzed with FAF, OCT, and pattern ERG (PERG), allowing a comparison between the parameters describing impairment of RPE and photoreceptors (Fig. 1). In a subgroup of patients (*n* = 14), fixation and retinal sensitivity to light were evaluated in mesopic conditions with a Nidek MP1 microperimetry (Nidek Technologies, Padova, Italy).

BCVA was measured using Snellen charts and transformed to the logMAR equivalent. A threshold of ≥1.0 logMAR was chosen to represent legal blindness. Kaplan–Meier survival analysis was performed using BCVA on the eye with a better VA. The area of definitely decreased autofluorescence (DDAF) was measured on FAF to represent the degree of RPE loss; the thickness of the outer nuclear layer (ONL) was measured on OCT to represent the degree of photoreceptor loss, and PERG amplitude was used to represent the degree of photoreceptor dysfunction in the macula. Additionally, full-field ERG amplitudes, reflecting peripheral retinal function, were evaluated and compared between the two groups. For the DDAF and ONL analyses, patients were masked to the graders. Detailed description on ERG, FAF, OCT, and microperimetry analyses are provided in the Supplementary Materials and Methods.

#### Genetic Analysis

Peripheral venous blood samples were obtained and genomic DNA was extracted from blood samples according to the standard procedure. Sequencing of the *ABCA4* gene in 13 patients was performed using Illumina Nextera Coding Exome capture protocol, with subsequent sequencing on Illumina NextSeq550 (Illumina, San Diego, CA, USA). In three patients, sequencing of the entire ABCA4 genomic locus was performed using single molecule molecular inversion probes library preparation and the Illumina NextSeq500 sequencing platform.^43^ The variants’ segregation with the disease in available families was analyzed by Sanger sequencing. In addition, Sanger sequencing was performed to confirm identified variants in two siblings.

#### Statistical Analyses

Data were analyzed using IBM SPSS Statistics software version 27.0 (IBM Corp. Armonk, NY, USA). The Mann–Whitney *U* test was used to compare median values of the measured parameters between the two patient groups, followed by multiple linear regression to account for the effect of age. Simple linear regression was used to study the effect of age on DDAF, PERG, and ONL, with age as the independent variable, whereas DDAF, PERG, and ONL were taken as dependent variables. A Spearman rank correlation test was adopted to assess whether there was a correlation between the degree of RPE atrophy (represented by the DDAF area) and loss of photoreceptors (represented by ONL thickness and PERG P50 amplitudes) within each genotype group. This process was followed by a multiple linear regression to explore genotype differences between ONL thickness and PERG P50 amplitudes for the same DDAF area. A Kaplan–Meier survival curve was used to determine the age when 50% of patients reached legal blindness. For this analysis, 4 of the 11 double null patients were excluded, because it was not possible to reliably determine the time when their BCVA decreased to ≥1.0 logMAR owing to a lack of BCVA data at early stages. Log-rank testing was used to test for statistical differences between the survival curves of the two patient groups. A *P* value of <0.05 was considered to indicate statistical significance.

For the comparison of median values between the two groups, the measurements around the time of the last ERG examination were used, because the ERG is performed least frequently owing to a high level of difficulty and time consumption and most other examinations were available at a similar time point. For correlations, examinations were considered to have been performed at a similar time point if done within 2 years of ERG testing. For one c.5714+5G>A patient, we excluded OCT data for this analysis because they were not performed within the 2 years from ERG examination. Longitudinal analysis of the DDAF area, ONL thickness, and PERG P50 amplitude was performed with all available data. There was good interocular symmetry in all patients and the right eye was used to compare the structural and functional parameters between the two groups.

### Data Availability

The data that support the findings of this study are available on request from the corresponding author A.F. or the first author J.S. The data are not publicly available because they contain information that could compromise research participant privacy.

## Results

### Ex Vivo Analysis: *ABCA4* Variant c.5714+5G>A Results in Partial Exon Skipping Retaining Approximately One-Half of Normally Spliced mRNA

**Figure 2.**
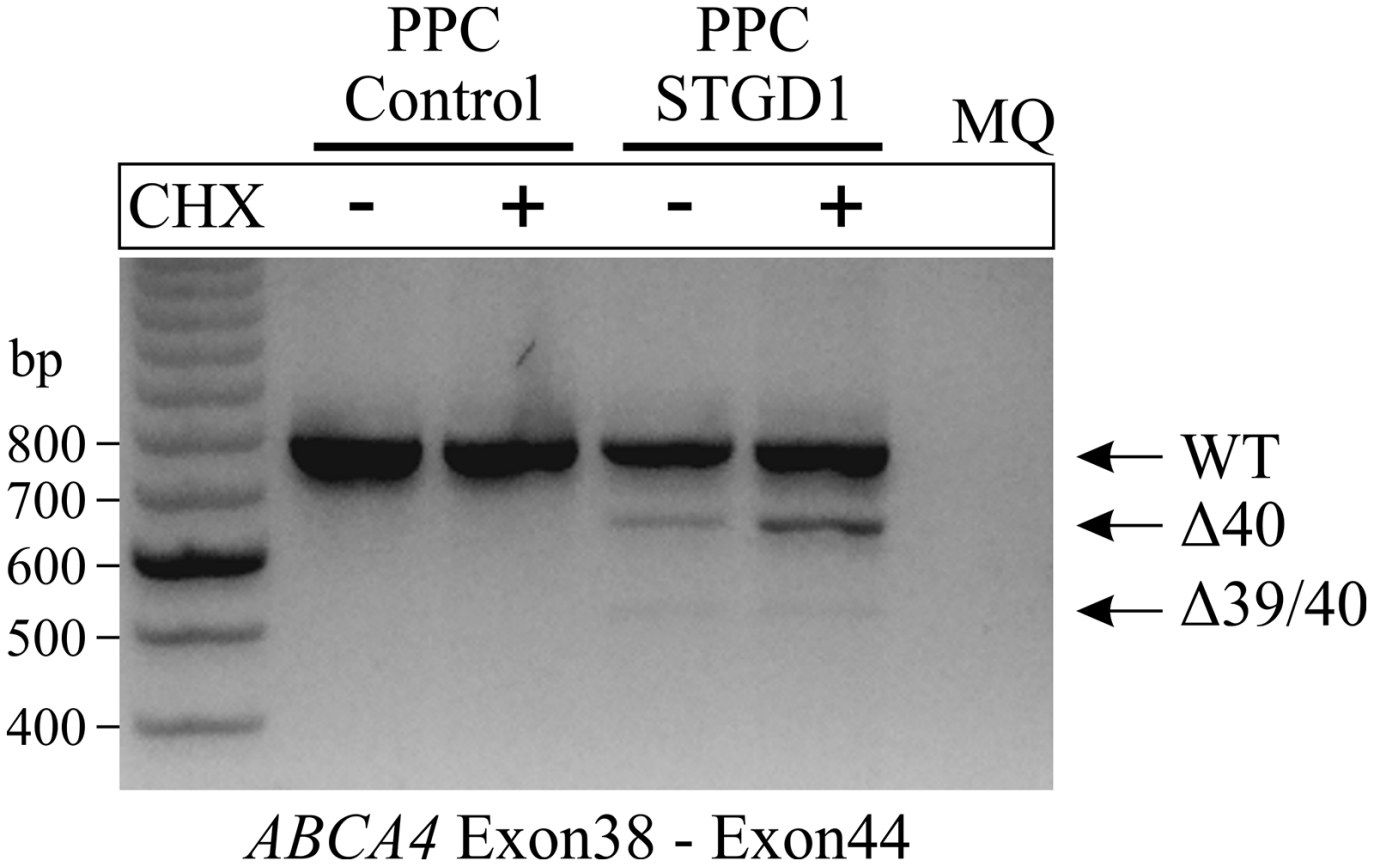
Gel image. PPCs enabled RNA analysis of the noncanonical splice site (NCSS) variant c.5714+5G>A. Control PPCs and PPCs from a patient with STGD1 were cultured without (–) or with (+) CHX that suppresses nonsense-mediated decay (NMD) owing to protein-truncating variants. RT-PCR using primers targeting exons 38 and 44 showed a 779-bp WT fragment, a 655-bp fragment corresponding with an exon 40 skipping, and a fragment of 525 bp corresponding with a combination of exon 39 and 40 skipping. The WT fragment band could be derived either from c.4539+1G>T allele, as NMD is incomplete, or from the correctly spliced product of the c.5714+5G>A allele. MQ, Milli-Q water; ∆, deletion.

To determine the effect of variant c.5714+5G>A on splicing in a retina-specific context, PPCs were generated from a STGD1 patient carrying c.5714+5G>A in *trans* with variant c.4539+1G>T. Variant c.4539+1G>T was thought to behave as null owing to its homozygous presence in persons with panretinal dystrophy.^30^ Moreover, two noncanonical splice site variants affecting the same splice donor site, c.4538A>G and c.4538A>C, when analyzed in vitro in human embryonic kidney (HEK293T) cells using midigenes, resulted in a 30-nt elongation of exon 30 and the skipping of exon 30, yielding the RNA product r.[4539_4540ins(30),4467_4539del] and the protein product p.[Arg1513_Arg1514ins10,Cys1490Glufs*12].^27^ RT-PCR analysis with primers located in exons 38 and 44 revealed the presence of products carrying RNA defects (Fig. 2). In particular, skipping of exon 40 on its own or in combination with exon 39 was observed. To better assess the amount of aberrant transcript, CHX treatment was performed to suppress the degradation of RNA products carrying protein-truncating mutations.

**Figure 3.**
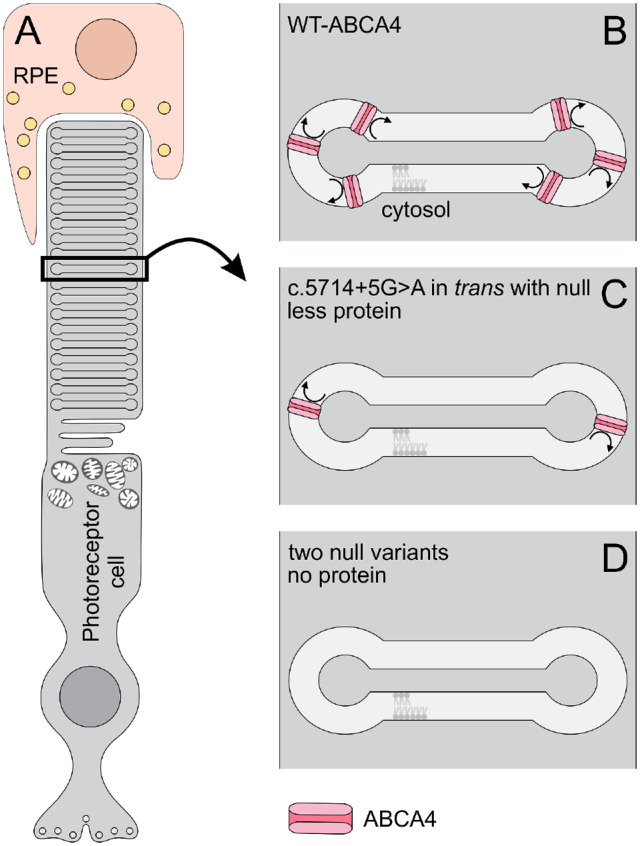
Schematic representation of the effect of the c.5714+5G>A allele. (**A**) RPE and photoreceptor cell with a black rectangle delineating an outer segment disc. (**B**) Normal number of ABCA4 transporters in a healthy person (**C**). Decreased number of ABCA4 transporters in a c.5714+5G>A patient. (**D**) Absence of ABCA4 transporters in a double null patient.

In the sample with no CHX treatment, the aberrantly spliced products amounted to 12.50% compared with 23.80% after CHX treatment. From 23.80% of mutant *ABCA4* mRNA treated with CHX, 20.95% of the product came from exon 39 skipping and 2.85% from a combination of exon 39 and 40 skipping, whereas 76.20% of the RT-PCR products were quantified as wild-type (WT). Because the WT fragment also contains the mRNA product of the c.4539+1G>T allele, we consider that 47.60% of mRNA from the c.5714+5G>A allele is mutant and 52.40% is WT (Fig. 3).

### Clinical Analyses

#### The c.5714+5G>A Patients Exhibit a Milder Phenotype Than Double Null Patients

**Figure 4.**
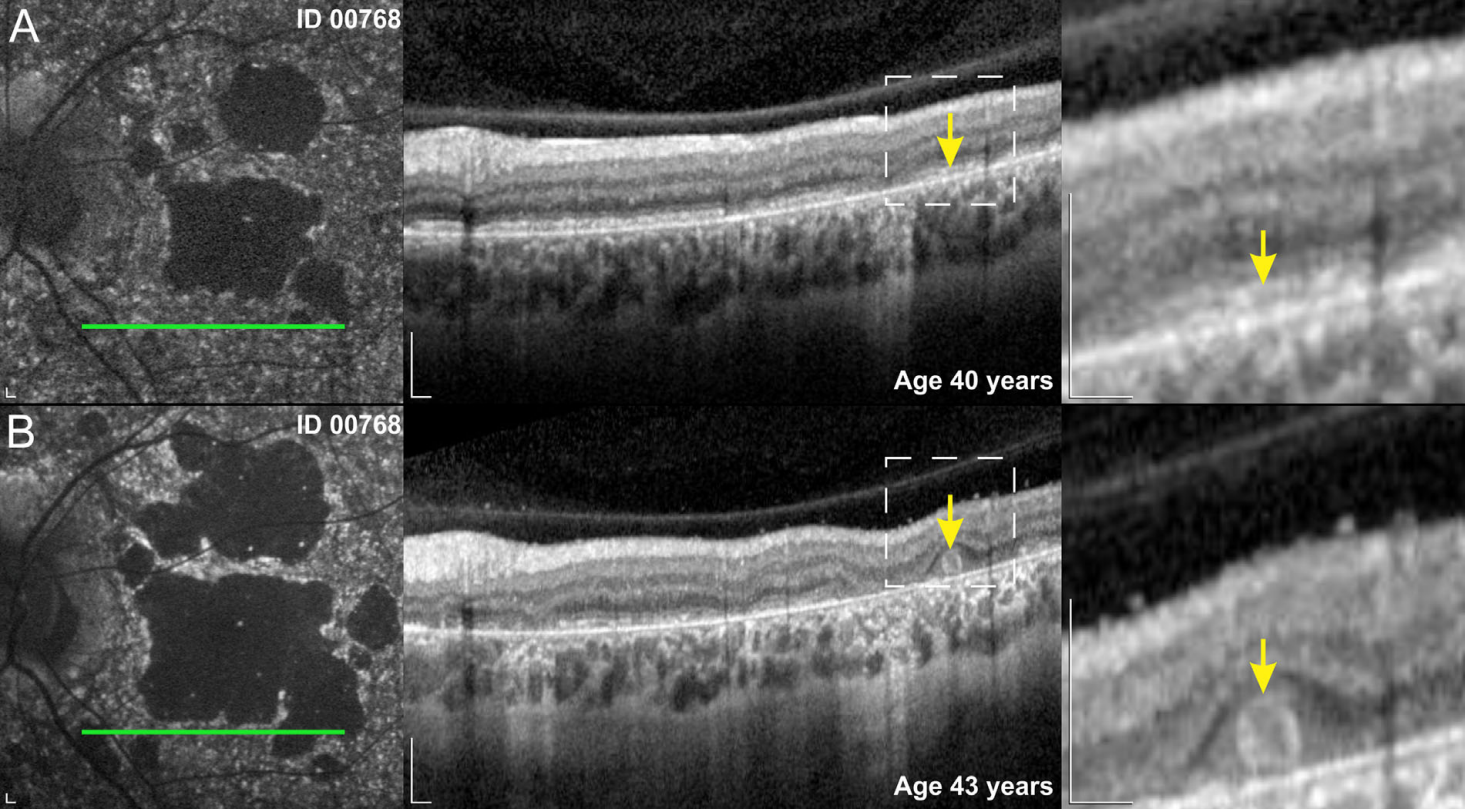
Outer nuclear tubulations. Tubulations in a c.5714+5G>A patient. Note the preserved RPE and photoreceptors layers (**A**), which transformed into photoreceptors grouping over the degenerating RPE in the period of 3 years (**B**). Scale bars, 200 µm.

**Figure 5.**
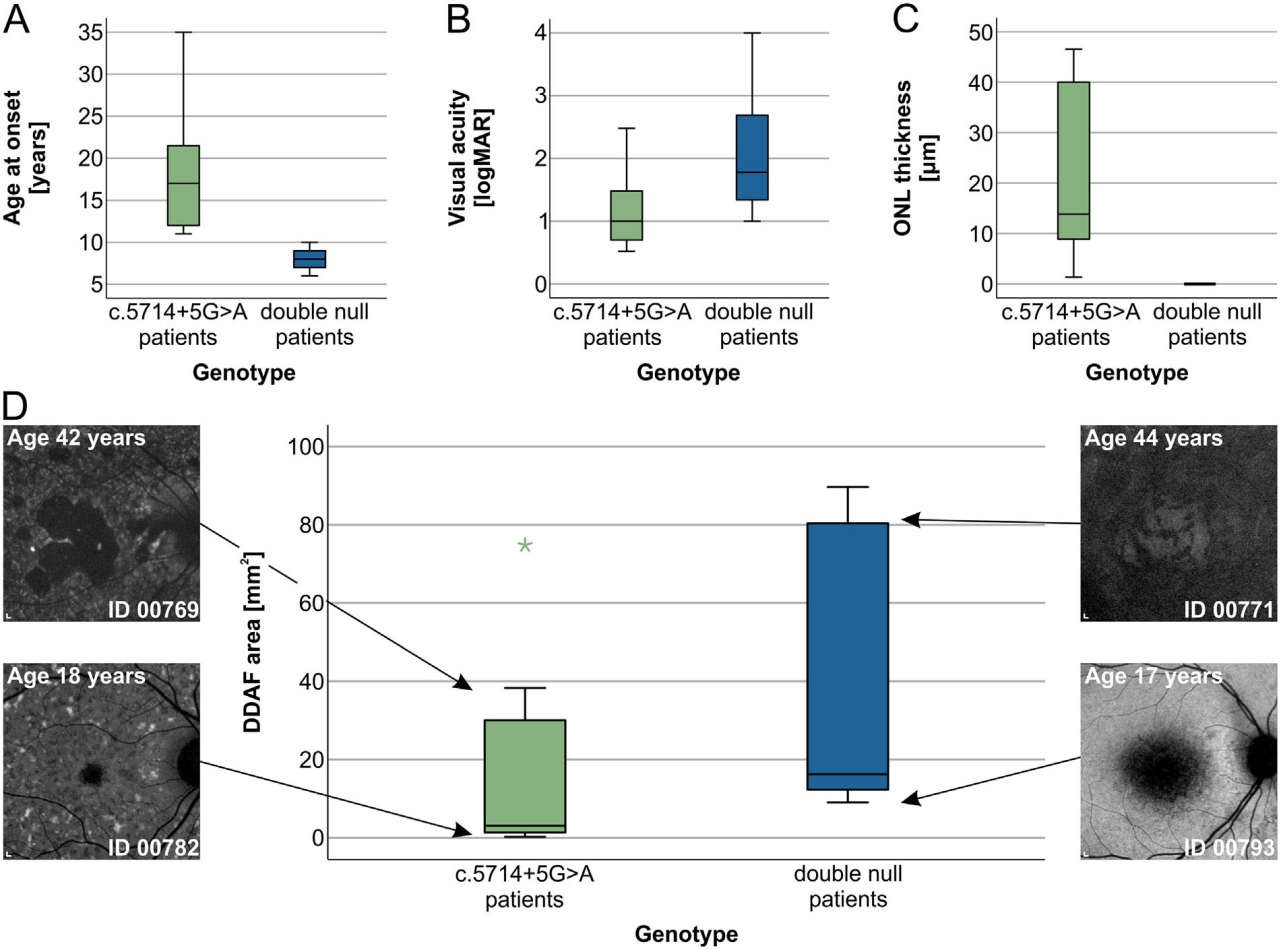
Boxplot charts showing differences between the two patient groups in the age of onset (**A**), VA (**B**), ONL thickness (**C**), and DDAF area (**D**). Horizontal lines represent median values, boxes half of the data and whiskers the remaining data except in the case of the outliers (*stars*). The c.5714+5G>A patients had significantly later age of onset, significantly better VA, thicker ONL and smaller DDAF areas than double null patients. (**D**) Representative FAF images of two pairs of similarly-aged patients from the two groups, shown on the left and right sides with their approximate position in the boxplot marked with arrows. Scale bars, 200 µm.

**Figure 6.**
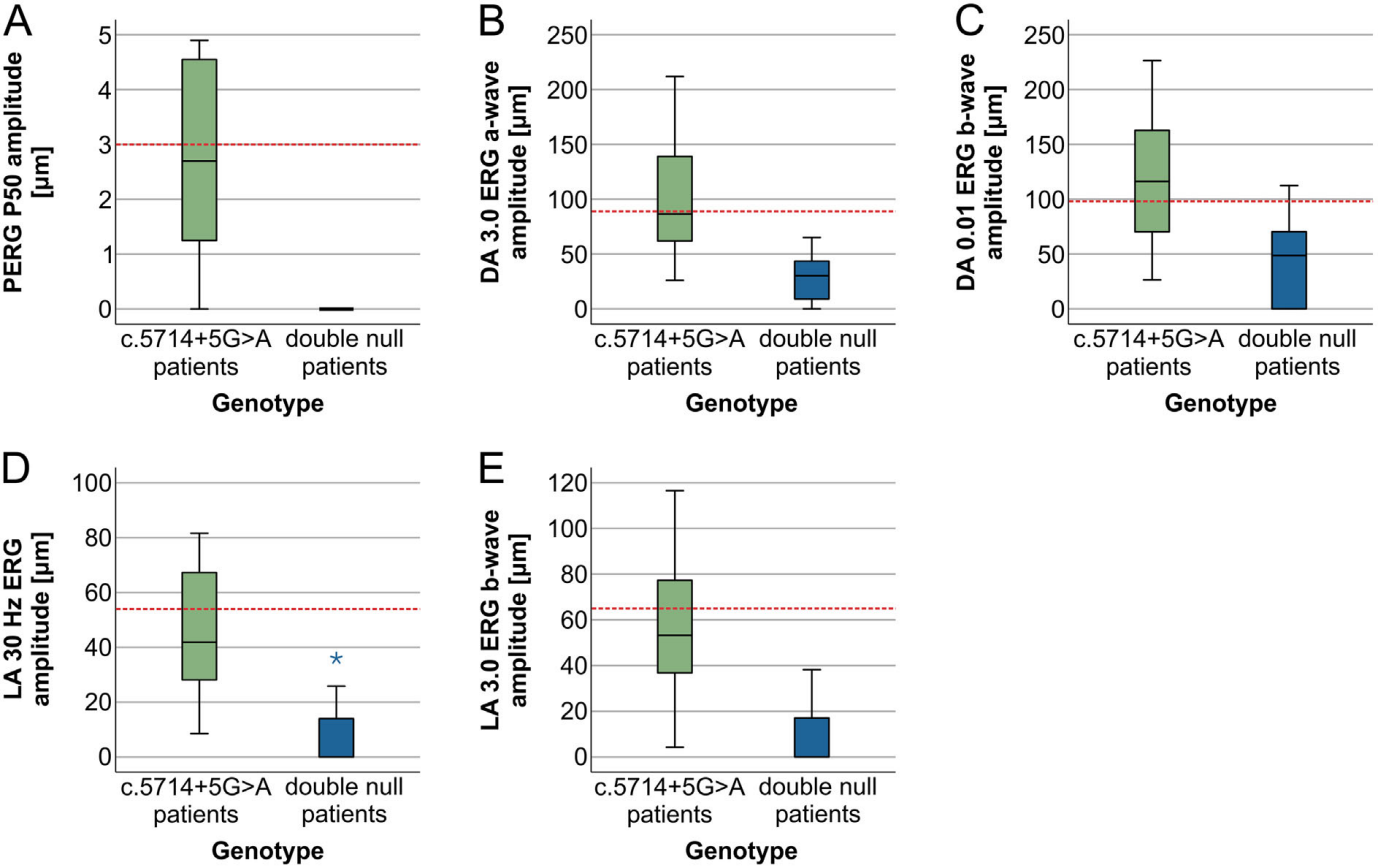
Boxplot charts showing the amplitudes of different ERG parameters in the two patient groups (**A**–**F**). Horizontal lines represent median values, boxes half of the data and whiskers the remaining data except in the case of the outliers (*stars*). Values were considered abnormal if they fell outside our laboratory normal range (marked with *red dashed line*). Note that, even though c.5714+5G>A patients were the same age or even older than double null patients, all their ERG responses were significantly better preserved, often within normal range.

The median values for all parameters and results of statistical comparison between the two groups are shown in the Supplementary Table S4 and represented graphically in Figures 5 and 6. The c.5714+5G>A patients had a significantly later median age of onset than double null patients (17 years vs. 8 years) (Fig. 4A). At the selected time point for analysis (at the time of the latest ERG examination), the c.5714+5G>A patients were relatively older than double null patients (34 years vs. 22 years), but had a significantly milder phenotype according to all measured parameters (Figs. 5 and 6, Supplementary Table S4). ONL was measurable in all c.5714+5G>A patients and absent in all double null patients (Fig. 5C). The PERG P50 amplitude was detectable above noise level in 6 of 7 c.5714+5G>A patients (86%) and undetectable in all double null patients (Fig. 6A). The c.5714+5G>A patients had significantly higher DA 3.0 ERG a-wave amplitude (Fig. 6B), DA 0.01 ERG b-wave amplitude (Fig. 6C), LA 30 Hz ERG amplitude (Fig. 6D), and LA 3.0 ERG b-wave amplitude (Fig. 6E).

Qualitative differences were observed in the FAF and OCT images between the two groups. In c.5714+5G>A patients, the regions of decreased FAF were more often scattered and had well-defined borders, whereas in double null patients they were typically centered in the macula and had poorly defined borders (representative images in Fig. 5D and Figs. 9C, 9F, 9I, 9L). On OCT, photoreceptor tubulations were observed in c.5714+5G>A patients but not double null patients (Fig. 4 and Supplementary Fig. S5).

#### The c.5714+5G>A Patients Exhibit a Delayed Decline of Structural and Functional Biomarkers in Comparison With Double Null Patients

**Figure 7.**
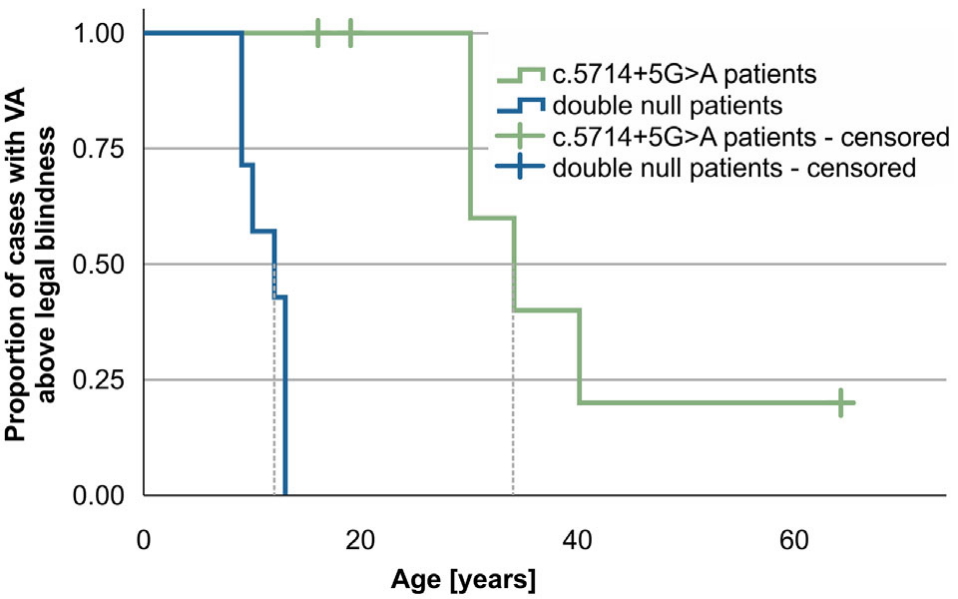
Kaplan–Meier survival analysis showing the ratio of patients reaching legal blindness (≥1.0 logMAR).

The Kaplan–Meier survival analysis predicted that the median age when 50% of the patients reached legal blindness was 34 years for c.5714+5G>A patients (95% confidence interval [CI], 25–43 years; *n* = 7) and 12 years for double null patients (95% CI, 7–17 years; *n* = 7). The difference between the groups was significant (log-rank test, *P* < 0.001) (Fig. 7).

Supplementary Table S5 shows the results of simple linear regression analysis, performed to determine the effect of age on PERG P50, ONL thickness and DDAF. In summary; in the c.5714+5G>A group, age significantly predicted the PERG P50 amplitude, ONL thickness, and DDAF area*.* In the double null group, age significantly predicted DDAF area. Their PERG P50 amplitude and ONL thickness were already undetectable at that time point (at the last ERG examination), precluding statistical analysis.

**Figure 8.**
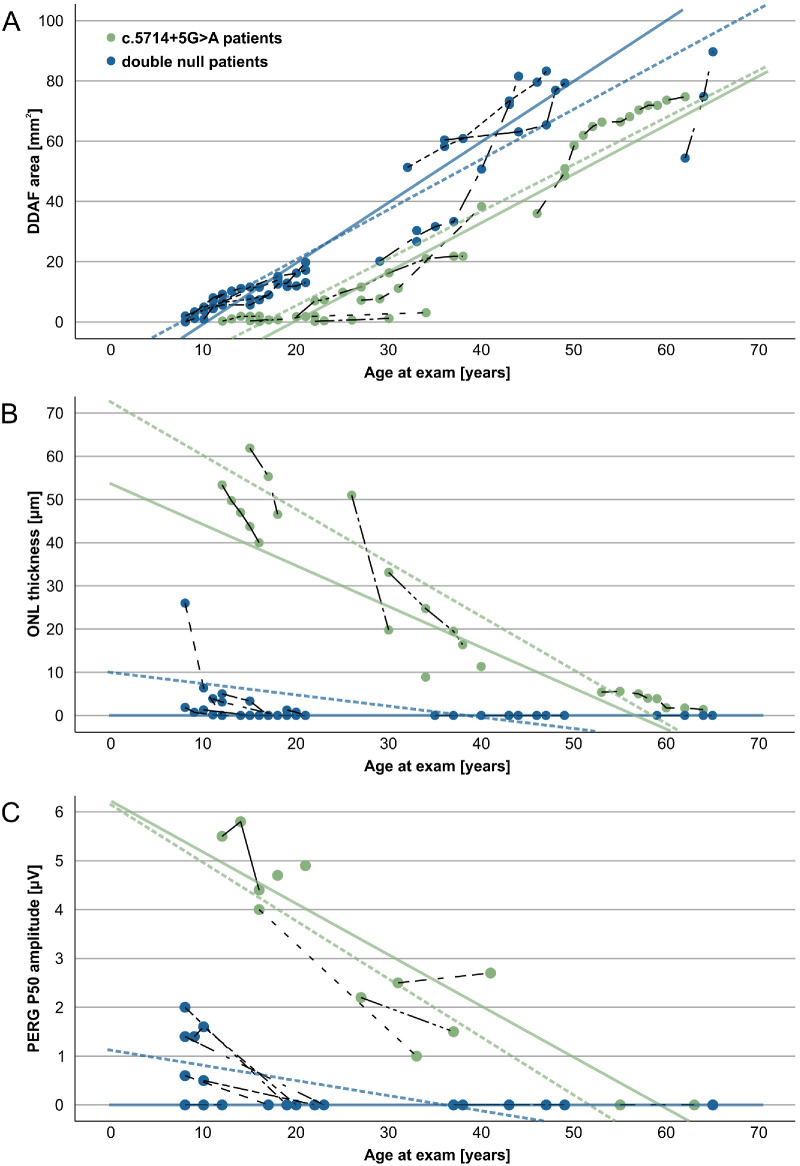
Longitudinal analysis of DDAF area (**A**), ONL thickness (**B**), and PERG P50 amplitude (**C**). Data points from individual patients are connected with *dashed interpolation lines*. The two *solid fit lines* are drawn using the data from exams around the last ERG testing. Dashed lines are drawn using the data from exams around the first ERG testing. The *green* represents the c.5714+5G>A patients and *blue* represents the double null patients. (**A**) Note a similar slope of DDAF enlargement, representing RPE loss, of the two groups, with an approximate delay of one decade in c.5714+5G>A patients (**B**, **C**). A greater time difference, of approximately four decades, was noted in parameters describing photoreceptors impairment.

The longitudinal analysis that included data from all time points showed detectable PERG50 and DDAF in double null patients and decline of all parameters with age in both patient groups (Fig. 8). The loss of RPE (DDAF area enlargement) was relatively linear in both groups, reaching extensive RPE loss with an approximate one decade difference (Fig. 8A), whereas parameters reflecting photoreceptor loss (ONL thickness and PERG P50 amplitude) decreased more rapidly in double null than c.5714+5G>A patients, reaching undetectable levels with a difference of approximately four decades (Figs. 8B and 8C).

#### The c.5714+5G>A Exhibit Different Ratios of RPE and Photoreceptor Damage Than Double Null Patients

**Figure 9.**
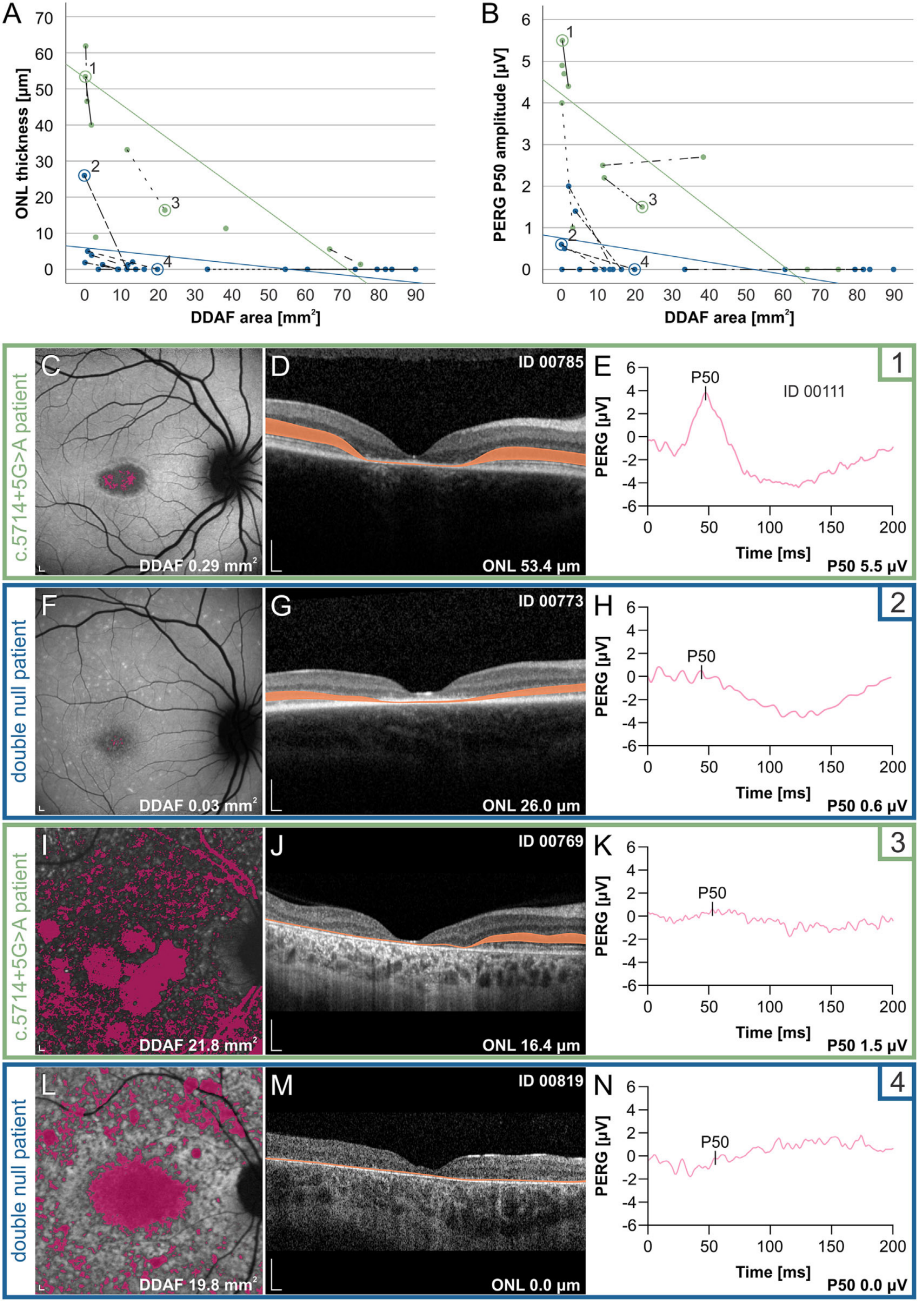
Different ratios of RPE and photoreceptor impairment in c.5714+5G>A patients (marked with *green*) and double null patients (marked with *blue*). (**A**) Correlation between ONL thickness and DDAF area. (**B**) Correlation between PERG P50 amplitude and DDAF area. Longitudinal data from the same patients are noted with *dashed lines*. Regression lines are drawn based on the first examination. (**C**–**N**) FAF, OCT scan through the fovea and PERG of representative patients with early and late stage of RPE atrophy. The individual patients are circled and marked with numbers 1 to 4 in (**A**) and (**B**). ONL is shown in orange on OCT images (**D**, **G, J, M**), and PERG P50 responses are shown in (**E**, **H**, **K**, **N**), whereas combined DDAF areas, shown in *pink*, are presented in (**C**, **F**, **I**, **L**). Note the relative preservation of ONL (**A**) and PERG P50 (**B**) of c.5714+5G>A patients across all DDAF sizes, most notable at the stage of minimal RPE atrophy, where double null patients already exhibited severe ONL and PERG P50 loss. In double null patients, a decreased ONL was observed even in the areas without notable RPE damage (RPE atrophy or extensive flecks) (**F**, **G, L, M**). Scale bars, 200 µm.

The correlations between the parameter reflecting RPE damage (DDAF), and the parameters reflecting photoreceptor damage (PERG and ONL) are shown in Figure 9. Multiple linear regression comparing the ratio of RPE versus photoreceptor damage between the two genotypic groups showed that, for the same DDAF area, c.5714+5G>A patients had significantly better preserved photoreceptor structure (thicker ONL) (B = 19.049; 95% CI, 7.675–30.423; β = 0.650; *P* = 0.003) and photoreceptor function (higher PERG P50 amplitudes) (B = 2.492; 95% CI, 1.246–3.737; β = 0.692; *P* = 0.001). Two representative patient pairs with similar DDAF areas are shown in Figures 9C–9N.

**Figure 10.**
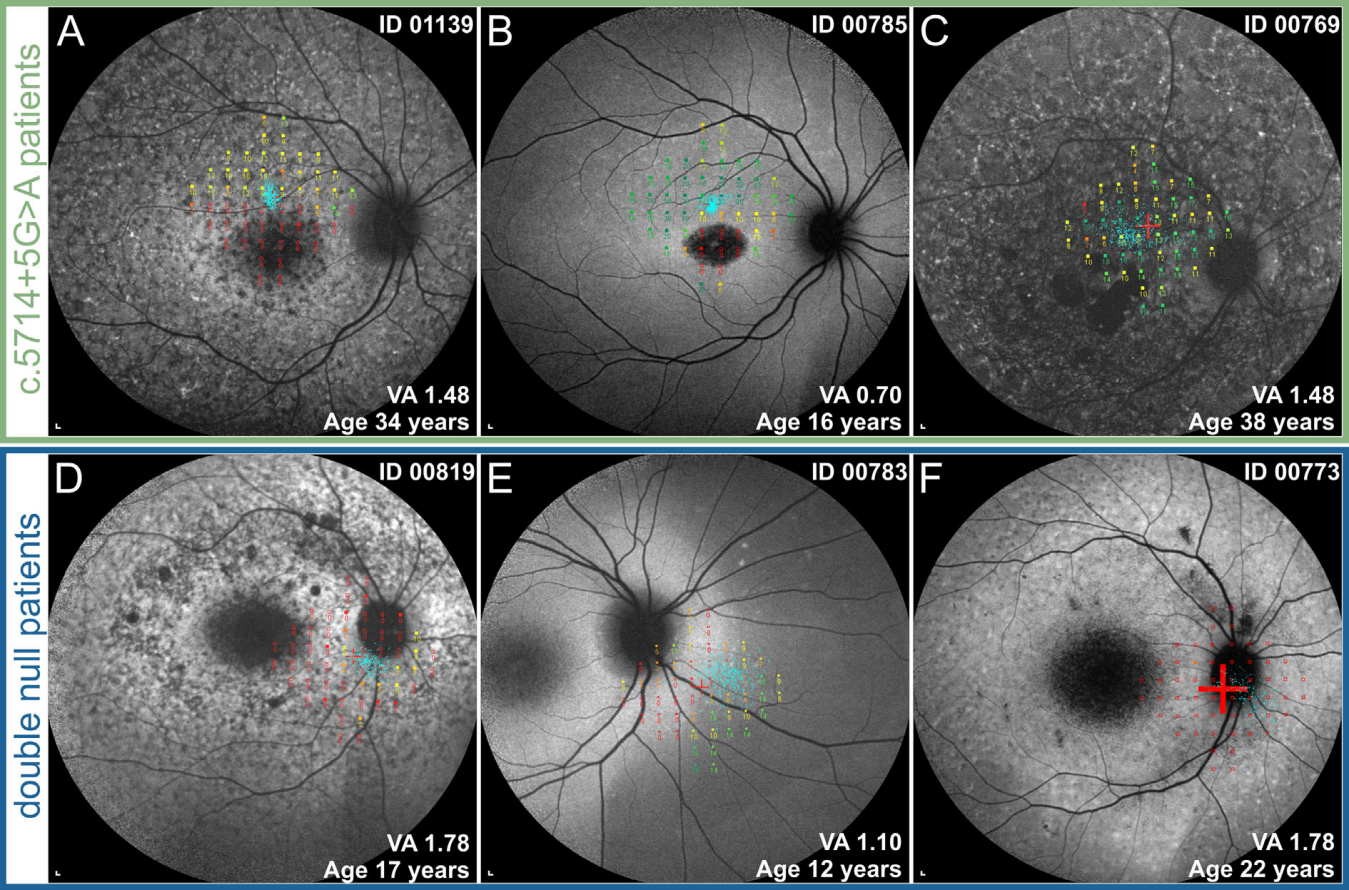
Microperimetry results superimposed over 55° FAF images in representative c.5714+5G>A patients (**A**–**C)** and double null patients (**D**–**F)**. Fixation points, determining a preferred retinal locus, are marked with *blue dots*, and a retinal sensitivity map of 56 tested loci is projected around the fixation points. The sensitivity of tested loci is expressed as a color ranging from *red* (0 dB—poor sensitivity) to *green* (20 dB—good sensitivity). Patients with c.5714+5G>A fixated on the border of the central FAF lesion and demonstrated preserved photoreceptor function in those areas.

Microperimetry was assessed in three representative patients from each group (Fig. 10). All three c.5714+5G>A patients exhibited good retinal sensitivity at the regions outside the central region of RPE atrophy and a shift of fixation locus near the border of the atrophy (Figs. 10A–C). All three double null patients exhibited severe loss of retinal sensitivity outside the central region of RPE atrophy and in all three cases the fixation shifted to the peripapillary region, far from the border of the atrophy (Figs. 10D–F).

The fixation of our double null patients (Figs. 10D, 10F) shifted far from the central lesion to the peripapillary region, indicating there is an extensive loss of photoreceptor function in the maculae of these patients. In (Fig. 10E), there is a double null patient with completely eccentric fixation, indicating even more extensive loss of photoreceptor function. Note the differences between different genotypes even in patient pairs with similar degree of RPE atrophy (represented by dark areas on FAF images) (Fig. 10A vs. Fig. 10D and Fig. 10B vs. Fig. 10E).

## Discussion

The study combined the approaches of in vitro and clinical analyses to determine the impact of c.5714+5G>A, one of the most frequent *ABCA4* variants. The PPC assay revealed that the c.5714+5G>A allele retains approximately one-half of the WT ABCA4 RNA and patients harboring the variant in trans with null (i.e., in isolation) exhibited signs suggestive of an RPE-first disease, contrasting the signs suggestive of photoreceptor-first disease observed in double null patients.

It was expected that disease associated with c.5714+5G>A, retaining some protein activity, would be milder that disease in double null patients. However, what is novel in this study is exploring the question whether the disease is milder evenly in terms of the degree of RPE and photoreceptor damage and, if yes, in what direction. This question arises from observations of different phenotypes associated with different mild alleles. For example, the phenotype associated with the mild allele p.(Gly1961Glu) is characterized by a primary foveal photoreceptor damage and minimal to absent RPE involvement (flecks).^9^^,^^44^^–^^46^ In contrast, other mild alleles often result in a widespread RPE disease and, in some cases, spared foveal photoreceptors.^9^^,^^46^ Specific examinations of phenotypes associated with different mild alleles can improve understanding of *ABCA4* disease, especially when correlated with their effects on a molecular level.

### Molecular Consequence of the c.5714+5G>A Variant

The mRNA from PPCs from a patient carrying variant c.5714+5G>A showed partially defective splicing, resulting in three RNA products, a normally spliced one and two truncated ones that presumably undergo nonsense-mediated decay. RT-PCR of mRNA from PPCs from a patient carrying c.4539+1G>T and c.5714+5G>A using primers in exons 38 and 44 showed a major normal product and minor exon 40 and exon 39/40 deletion products. Quantification of the RT-PCR products showed 52.40% WT and 47.60% mutant *ABCA4* mRNA, which assumes that nonsense-mediated decay suppression is complete in CHX-treated cells. The PPC results (52.4% WT), given that these retinal-like cells better represent the in vivo situation,^29^ partially confirm the results of the midigene-based splice assay.^27^ These data suggest that c.5714+5G>A should be considered to behave at the boundary between mild and moderately severe.

Our results suggest that the molecular consequences of the c.5714+5G>A variant are a decrease in the abundance of normally spliced *ABCA4* mRNA owing to a partially defective splicing and, therefore, a lower quantity of the ABCA4 transporters (Fig. 4). It remains to be seen whether there are any splicing differences between different retinal cell types, such as between photoreceptor cells and RPE cells.

### Clinical Consequences of the c.5714+5G>A Variant and Implications for Disease Pathogenesis

Compared with double null patients, c.5714+5G>A patients exhibited a milder phenotype, as is expected for an allele conferring residual function.^9^^,^^47^ Although this finding was not surprising, the analysis of biomarkers representing RPE and photoreceptor damage revealed intriguing findings that suggested genotype-dependent differences in the sequence of events of disease pathogenesis. Double null patients exhibited early and severe photoreceptor loss (i.e., photoreceptor-first disease), which is in concordance with previous reports.^13^^,^^21^^,^^48^ In contrast, the clinical appearance of c.5714+5G>A patients suggested a RPE-first sequence of events, with photoreceptor loss occurring secondarily.

Three clinical observations support this premise:

i) A lower ratio of photoreceptor versus RPE damage in c.5714+5G>A patients. Multiple regression analysis accounting for the size of RPE atrophy revealed significantly better-preserved photoreceptor structure and function in c.5714+5G>A patients. The c.5714+5G>A patients exhibited photoreceptor preservation even in the maculas with extensive RPE atrophy, whereas the double null patients exhibited severe photoreceptor impairment, even in cases with little RPE atrophy and outside of the main RPE lesion. Different ages at onset and, with that, different disease stages, are unlikely a major factor affecting the observed differences in photoreceptor preservation because differences were evident even in patient pairs with smaller degrees of RPE loss in earlier stages of the disease (Figs. 9 and 10).
ii) Microperimetry results. It has been previously shown that after the loss of foveal cones the preferred retinal locus of fixation shifts to the area with best photoreceptor preservation.^49^ Looking at microperimetry data of patient pairs with similar stage of disease in terms of RPE damage, the preferred retinal locus of fixation of c.5714+5G>A patients located next to the border of RPE atrophy, suggesting viable photoreceptors above the preserved RPE, whereas the preferred retinal locus of fixation of double null patients is located significantly further out, indicating significant photoreceptor loss above the preserved RPE (Fig. 10).
iii) Presence of photoreceptor tubulations in c.5714+5G>A but not double null patients. Although the RPE does not depend on the presence of photoreceptors, photoreceptors cannot survive without the RPE.^50^ Although the meaning of photoreceptor tubulations is not completely understood,^51^^,^^52^ they most likely represent a short transitional period before photoreceptor degeneration in diseases that primarily affect the RPE.^53^^–^^55^ Outer retinal tubulations have previously been described in patients with STGD1 and were thought to represent primary RPE loss with viable photoreceptors grouping over the degenerating RPE.^16^^,^^51^^,^^56^ In the present study, tubulations have been observed in c.5714+5G>A, but not double null patients (Fig. 4 and Supplementary Fig. S5).

It has been proposed that ABCA4 also localizes in the RPE^57^; however, the different sequence of events observed in the two patients groups is likely not a reflection of diseases of separate cells types. The RPE-first sequence of events had been proposed previously as the main hypothesis behind *ABCA4* disease pathogenesis and is thought to be caused by primary RPE apoptosis after phagocytosis of bisretinoid-laden photoreceptor outer segments, followed by secondary photoreceptor degeneration.^7^^,^^58^^–^^61^ The present study supports this hypothesis in association with the c.5714+5G>A variant. In contrast, double null patients in this and previous studies^13^ exhibited signs of severe photoreceptor loss preceding RPE loss.

The results of the in vitro analysis showing defective splicing may provide some insight into the reasons behind the RPE-first sequence of events. The latter revealed defecting splicing leaving approximately 50% of normal RNA. We propose that the presence of functional ABCA4 transporters in the photoreceptor outer segments spare the photoreceptors from the primary degeneration, perhaps by preventing the accumulation of toxic bisretinoids on the cone outer membranes.

Unfortunately, although the timeline and the sequence of events were different, the end result in both patient groups was a severe cone–rod dystrophy; therefore, it seems that the remaining ABCA4 protein from one c.5714+5G>A allele is not enough to prevent panretinal degeneration. The results of this study relate to the emerging gene replacement therapy, which will act in the same way, that is, by increasing the number of ABCA4 transporters. It is, therefore, of interest to determine the level of gene expression needed to save the retina from developing disease.

Further genotype–phenotype studies are needed to determine whether our observations are variant specific or could be extrapolated to other *ABCA4* variants that result in the decreased number of functional ABCA4 transporters. Other noncanonical splice site variants in *ABCA4* have also been shown to result in partially defective splicing,^27^ including c.5196+1137G>A, for which the PPC assay showed approximately 55% to 60% of the normally spliced product; however, a detailed analysis of biomarkers reflecting photoreceptor and RPE loss was not performed.^47^ It is important to note that the terms RPE-first and photoreceptor-first may be oversimplified. It is more likely that there are RPE-damaging and photoreceptor-damaging processes that occur simultaneously, but at a different ratio in each patient, thus, resulting in the known variability of STGD1 disease.

Other mild alleles may result in more complex effects on disease pathways, especially those affecting protein structure, its localization, and/or function. For example, biochemical experiments suggest that p.(Gly1961Glu) alters the transretinal related activity of the ABCA4 transporter,^62^ which may be related to its specific phenotype.^9^^,^^44^ Other variants that result in mislocalization have been proposed to add an additional toxic effect to the retina.^63^

### Classification of the c.5714+5G>A Variant

Based on RNA spliced defects observed in the transfected HEK293T cells or patient-derived PPCs, the severity assessment of variants in the *ABCA4* gene can be made. Variants that showed >30% and ≤70% correct RNA were previously classified as variants with a moderate effect,^8^ and this cohort would include c.5714+5G>A with 52.40% of correct RNA. Additional modelling studies (FPMC, personal communication) indicate that variants with a moderate effect are associated with >20 and ≤40% of the remaining ABCA4 activity, which puts c.5714+5G>A variant in the mild category. However, it should be considered, that PPCs do not fully represent the in vivo retinal situation. In fact, the gene marker analysis (Supplementary Fig. S4) showed that the patient-derived PPCs have more rod–photoreceptor and RPE cell characteristics rather than cone–photoreceptor characteristics. Additional studies of this kind are, thus, warranted. In vitro data overall have to be interpreted with caution, because they often conflict with clinical data in *ABCA4*-associated retinopathy. For example, the p.(Gly1961Glu) variant has severely abnormal activity in functional studies,^62^ but is very mild and incompletely penetrant in genetic and clinical studies.^44^^–^^46^ Nevertheless, the retained normally spliced product of c.5714+5G>A is not expected to have any functional or localization abnormalities; thus, the results may be more reliable.

### Study Strengths and Limitations

The study's main strengths were the use the combined in vitro and clinical approach focused on a single *ABCA4* variant, which enabled us to provide a comprehensive overview of its impact. The PPCs provided the milieu that more closely resembled the in vivo situation and the clinical part of the study included the largest genetically homogenous group of c.5714+5G>A patients so far. Moreover, quantitative analysis if DDAF, ONL, and PERG from a very similar area of the central retina allowed a precise evaluation of RPE and photoreceptor damage in patients with different genotypes. Although clinical descriptions of c.5714+5G>A were published before, they included smaller groups of patients^9^^,^^13^^,^^30^ with compound heterozygous genotypes,^30^ and mostly qualitative assessment of ERG, FAF, images, SD-OCT images,^9^ or limited analysis of selected parameters.^13^

The limitations of the study are mostly related to the retrospective study design and low patient numbers. Although the phenotype of each group was specific enough that we were able to show significant differences for all measured parameters, a larger cohort would substantiate and strengthen our claims. Another limitation was the limitation of the Heidelberg Spectralis software, as it does not enable segmentation through the whole 30° × 30° area; thus, quantitative measurements of the ONL was only possible in the region within the Early Treatment of Diabetic Retinopathy Study (ETDRS) retinal grid, which was used to represent the ONL damage in the macula. It would be of interest to quantify the ONL outside the ETDRS area, where FAF is more often preserved; however, this factor was outside the scope of the present study. In double null patients, the qualitative analysis of ONL outside the ETDRS area showed reduced or absent photoreceptors even in the regions with preserved FAF signal (e.g., Figs. 9F, 9G, 9L, and 9M). In contrast, in c.5714+5G>A patients, photoreceptors were mainly preserved in the regions with preserved FAF signal (e.g., Figs. 9C, 9D, 9I, and 9J). Furthermore, the degree of photoreceptor impairment outside the ETDRS grid was corroborated by functional PERG and microperimetry analysis, which covered larger parts of the macula. The third limitation was that we did not measure early signs of RPE damage, but only end-stage RPE loss, that is, the DDAF area. Hyperautofluorescence quantified using quantitative FAF method has been extensively studied by Sparrow et al.^64^ as a marker of early RPE damage; however, this measurement requires a special sensor, which is not available at our institution. Another parameter, questionably decreased autofluorescence has also been proposed as a biomarker of RPE damage, representing a transition zone between the healthy retina and DDAF,^65^ but is very difficult to reliably quantify on FAF images. In contrast, the DDAF area, which represents RPE loss, has been defined as the most reliable biomarker for following *ABCA4* disease progression.^65^^–^^69^

## Conclusions

We determined the effect of variant c.5714+5G>A on splicing in patient-derived PPCs, which suggested that this is a variant with a mild to moderate impact. This finding was consistent with the clinical part of the study, showing a significantly milder phenotype in c.5714+5G>A than in double null patients. Moreover, the differences in ratios of RPE and photoreceptor damage between the two patient groups suggested genotype-dependent disease pathogenesis. Although a complete absence of the ABCA4 transporter would likely result in a photoreceptor-first disease, the presence of a decreased number of ABCA4 transporters seemingly shifts the pathogenesis toward RPE-first disease. Additional studies with larger groups of patients need to be done to confirm our observations.

## Supplementary Material

Supplement 1

Supplement 2

Supplement 3

Supplement 4

Supplement 5

Supplement 6

Supplement 7
