# Supplementary material for: ABCA4 Variant c.5714+5G>A in Trans With Null Alleles Results in Primary RPE Damage
Source: Invest Ophthalmol Vis Sci. 2023 Sep 20;64(12):33. doi: 10.1167/iovs.64.12.33 (PMC10516765; doi:10.1167/iovs.64.12.33)
Supplement: Supplement 1 [file iovs-64-12-33_s001.pdf]

Supplementary Figures

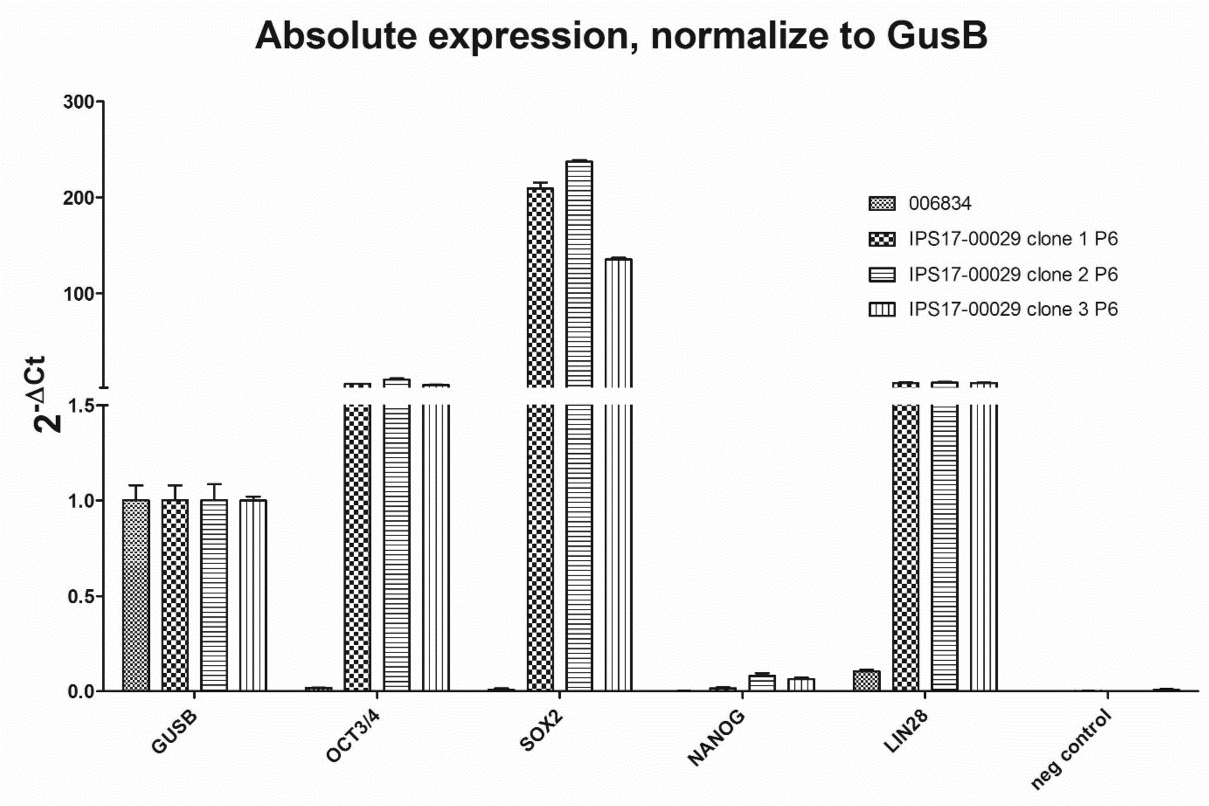

Figure S1. Gene expression of four genes used to reprogram Epstein-Barr virus (EBV)-immortalised cells into three induced pluripotent stem cell (iPSC) clones, normalised to *GUSB*, compared to the parental EBV-immortalised cell line (ΔCt). Ct values are normalized with the housekeeping gene *GUSB.*

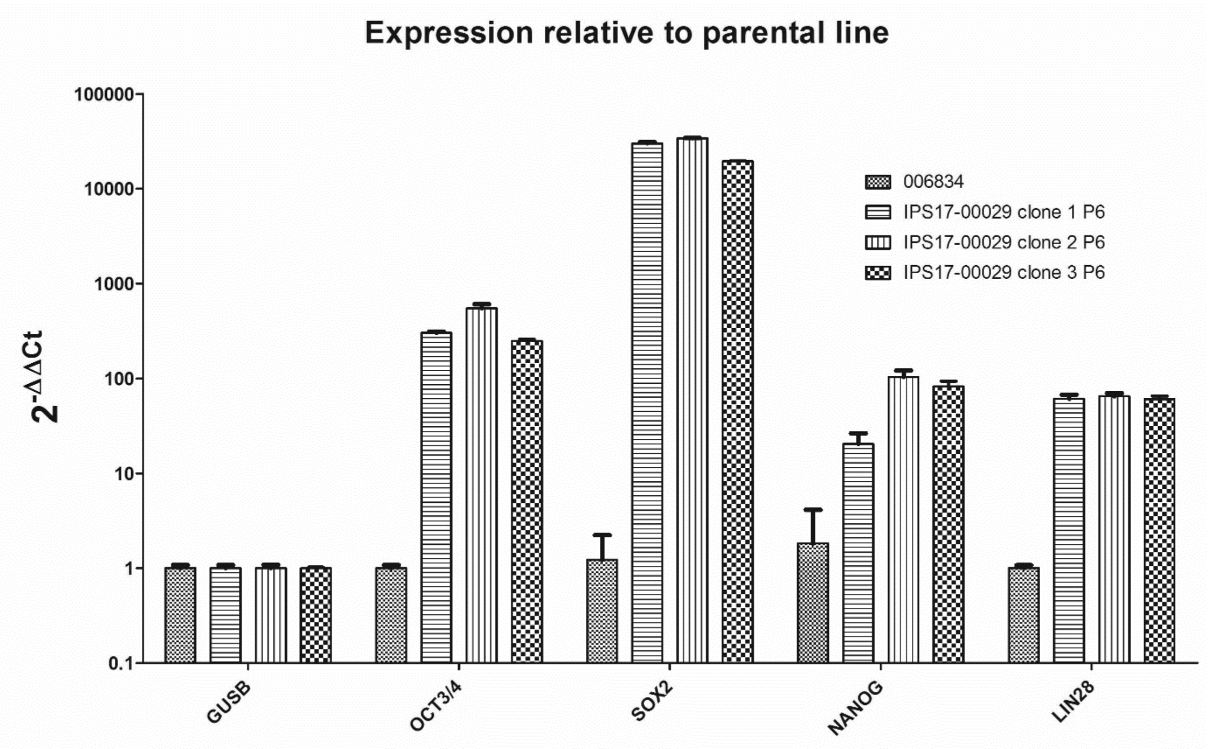

Figure S2. Pluripotency gene upregulation after reprogramming (ΔΔCt). The expression fold difference of the iPSC clones is relative to the parental EBV cell line. Ct values are normalized with the housekeeping gene *GUSB.*

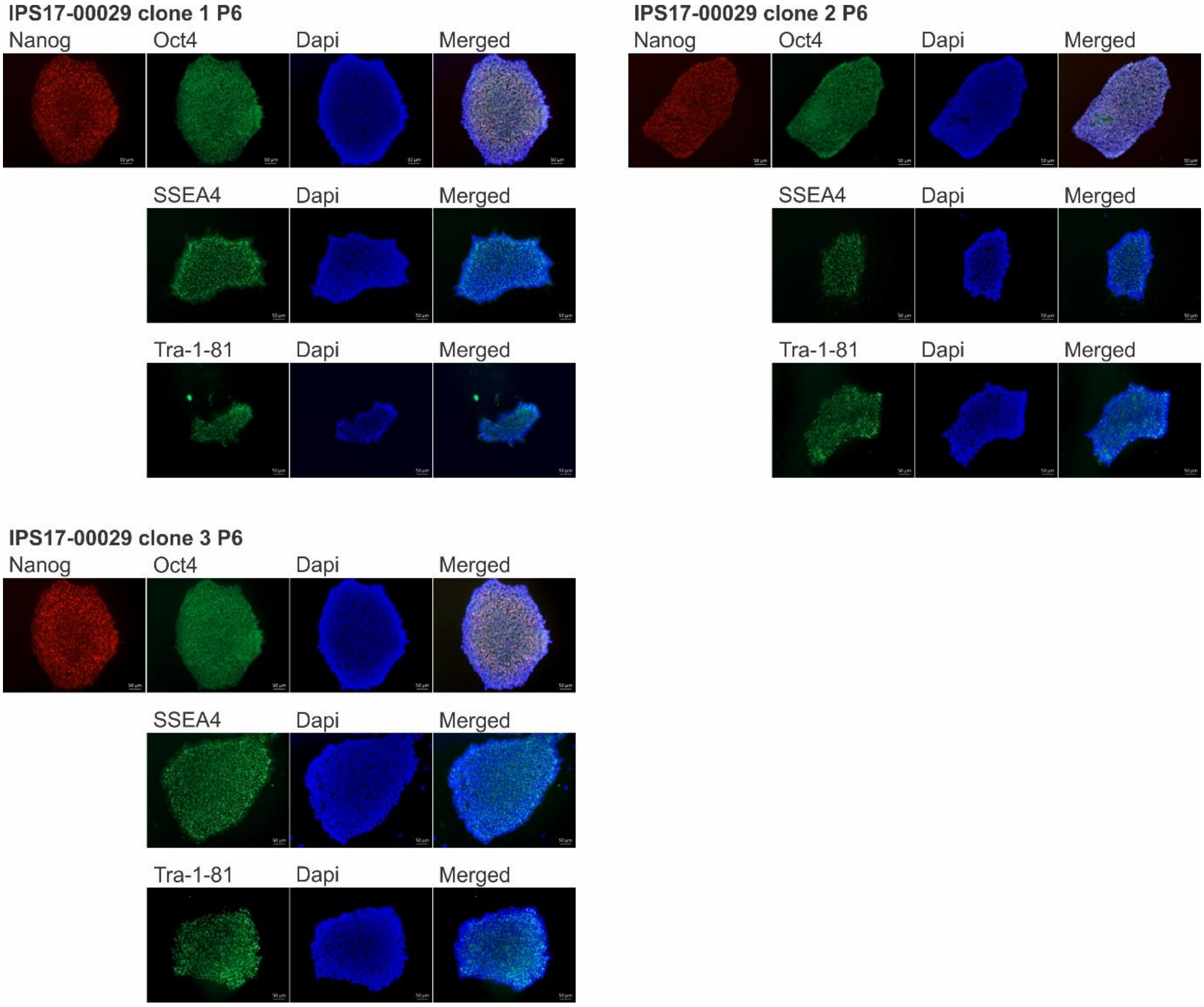

Figure S3. Immunofluorescence staining of the iPSC clones with pluripotency markers. All markers are clearly expressed.

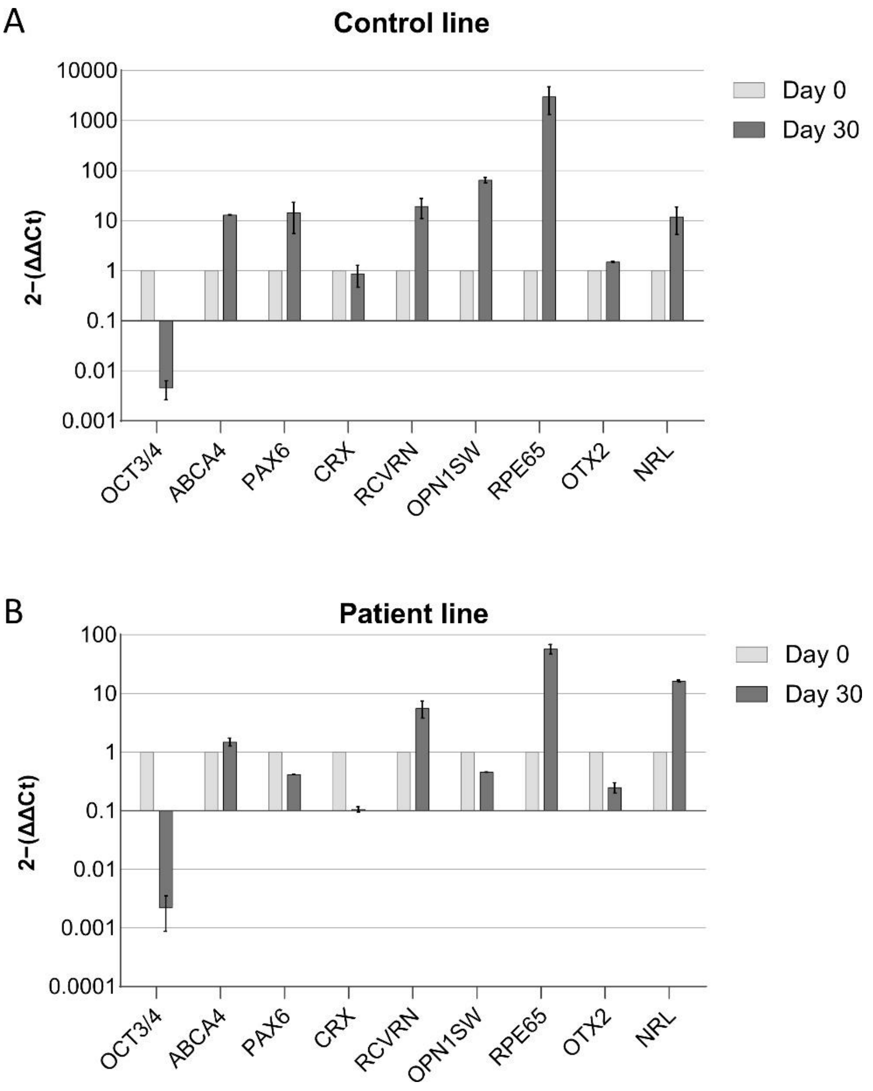

Figure S4. Retinal gene expression profiles of photoreceptor precursor cells (PPCs). The graphs show the average gene expression profile of control and patient derived iPSCs at day 0 (undifferentiated iPSC) and day 30 (differentiated to PPCs) of the differentiation protocol. For the patient line clone 3 is reported as the most representative. Ct values are normalized with the housekeeping gene *GUSB* and the expression of the markers in the PPCs was compared with the expression in the undifferentiated iPSCs*.* Expression profiles between the control and patient lines show variability but overall denote a trend of ongoing differentiation from iPSC to PPCs in both lines. (**A**) In the control line, expression of the pluripotency marker *OCT3/4* was significantly decreased after 30 days of differentiation. Expression of the early retinal

marker *PAX6* was increased, while *OTX2* remained comparable between iPSC and PPCs. The photoreceptor progenitor markers *NRL* and *RCVRN* were increased after 30 days while *CRX* remained at the same level. Increased levels of *OPN1SW, ABCA4* and *RPE65* indicate presence of photoreceptor cells at later stages of differentiation and RPE. (**B**) In the patient line, expression of the pluripotency marker *OCT3/4* was significantly decreased after 30 days of differentiation. Early-stage differentiation markers such as *PAX6* and *OTX2* were decreased. The photoreceptor progenitor markers *NRL* and *RCVRN* showed higher levels of expression after 30 days, while levels of *CRX* were lower. The significant increased level of *RPE65* mRNA and the slight increased expression of *ABCA4* mRNA indicate the presence of both RPE cells and photoreceptor cells, respectively, at later stages of differentiation. Expression levels of *OPN1SW* were slightly decreased at 30 days of differentiation.

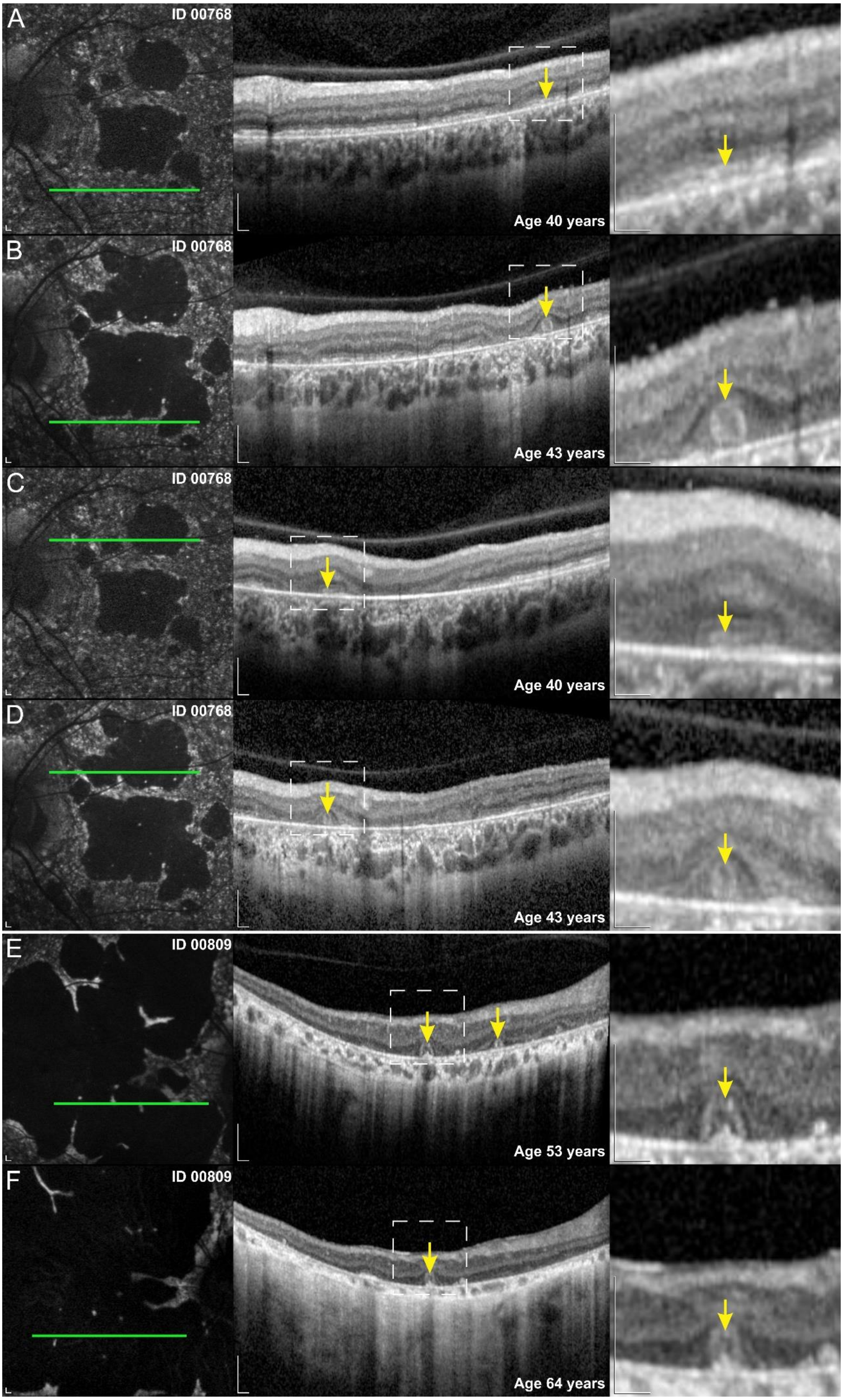

A
ID 00768
Age 40 years
B
ID 00768
Age 43 years
C
ID 00768
Age 40 years
D
ID 00768
Age 43 years
E
ID 00809
Age 53 years
F
ID 00809
Age 64 years

Figure S5. . Outer retinal tubulations. Tubulations in two c.5714+5G>A patients as the early signs of RPE degeneration. In the first patient, preserved RPE and photoreceptors layers (**A** and **C**) transformed into viable photoreceptors grouping over the degenerating RPE (**B** and **D**). In the second patients, tubulations persisted over the period of 11 years. Scale bars: 200 µm.

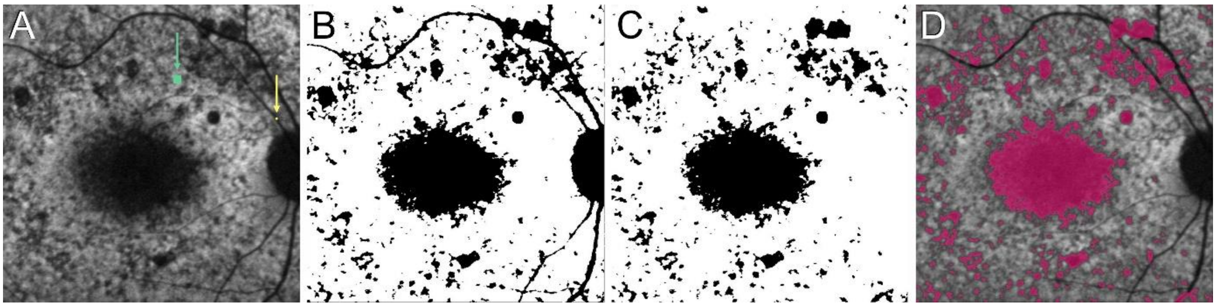

FIGURE S6. Analysis of DDAF area. (**A**) On 30° FAF image, DDAF was defined as being at least 90% black in reference to blood vessels (yellow square of 7 x 7 pixels area). The opposite reference point (green square of 21 x 21 pixels area) represented a healthy retina. (**B**) To easily grade the level of darkness, an original grayscale FAF image was converted to a binary image. Optic nerve and blood vessels were then manually eliminated (**C**) to not be included in the final step, where a combined DDAF area, shown in pink, was automatically calculated (**D**).

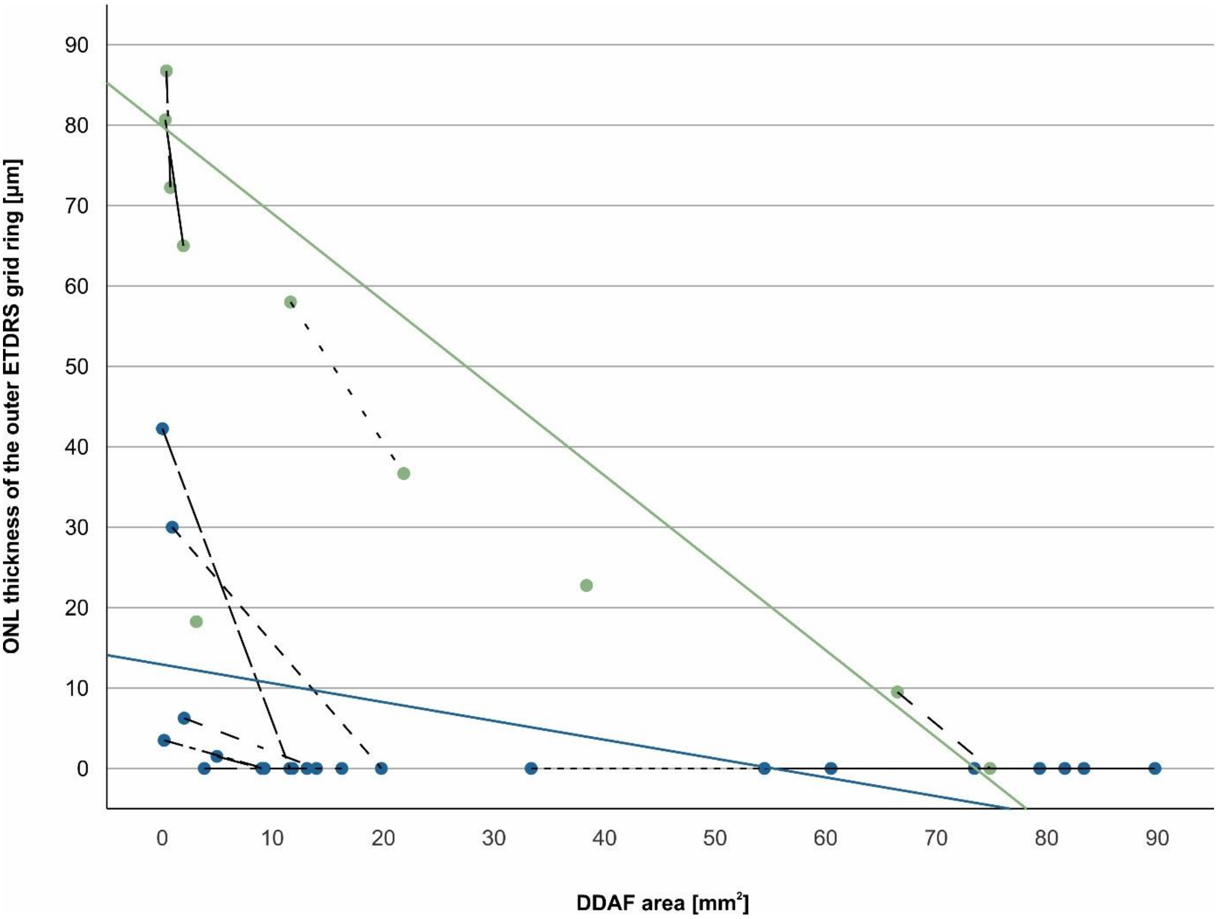

Figure S7. Correlation between DDAF area and ONL thickness of the outer ETDRS grid ring. ONL thickness of the outer ETDRS grid ring was analysed to evaluate ONL outside central DDAF. The results are similar to the mean thickness of all nine ETDRS grid zones.
