# Supplementary material for: ABCA4 Variant c.5714+5G>A in Trans With Null Alleles Results in Primary RPE Damage
Source: Invest Ophthalmol Vis Sci. 2023 Sep 20;64(12):33. doi: 10.1167/iovs.64.12.33 (PMC10516765; doi:10.1167/iovs.64.12.33)
Supplement: Supplement 2 [file iovs-64-12-33_s002.pdf]

Supplementary Materials and Methods

Analysis of the Area of the Definitely Decreased Autofluorescence

Fundus autofluorescence (FAF) images of the macula ware taken using Heidelberg Spectralis (Heidelberg Engineering, Heidelberg, Germany) with 488 nm excitation light and 500-700 nm emission filter. Images were processed using our own custom written codes in Matlab (The MathWorks, Inc., Natick, Massachusetts, USA) as follows (Figure S6): consecutive images from the same patient taken in different years were manually aligned using blood vessels and optic disk as reference. As FAF images were taken with different degree objective lenses on different occasions (either 55° or 30°), all images were cropped to 30° (Figure S6A). If possible, consecutive images were aligned. The manual alignment included only rigid transformations: rotation and horizontal and vertical translation without scaling or shearing. Images were filtered with 2 x 2 pixels median filter to reduce noise, and corrected for uneven illumination. The extent of retinal pigment epithelium (RPE) atrophy was estimated from definitely decreased autofluorescence (DDAF) area, which was determined according to ProgStar criteria [1] with 0% darkness reference set to the healthy retina and 100% darkness set to optic disc or main blood vessels, DDAF was calculated as the area with >90% darkness (Figure S6A).

Two researchers manually selected and agreed upon the reference points (J.S. and A.M.), while a single grader (A.M.) performed further analysis. To exclude random image noise the average grey value was calculated from an area of 21 x 21 pixels for healthy retina and on a smaller area of 7 x 7 pixels for black reference. The size of black reference area was smaller than the diameter of vessels. For black reference point, main blood vessel near optic disc or optic disc was chosen. Choosing the reference point for healthy retina was, however, more challenging. In some cases, we

had to use optical coherence tomography (OCT) images to help us find the unaffected part of the retina. In cases with almost compete atrophy on the 30° image, we found the grey reference point on 55° images. As fundus abnormalities increase with age, the positions of both reference areas were determined on the latest image and if possible, the same positions were chosen on all prior images. Grayscale FAF image was then converted to a binary image by replacing all pixels with luminance less than 90% of black reference with 0 (black) and all other pixels with 1 (white) (Figure S6B). Optic nerve and blood vessels were excluded manually (Figure S6C). All vessels adjacent to atrophic areas were manually excluded from the area calculation. However, vessels that are passing through the atrophic areas were not erased. Black pixels were then automatically summated (Figure S6D) and the area converted into square millimetres (mm^2). The internal reference was used for determination of single image pixel size in the majority of cases. When not, we used scale bar on the image. The described image analysis algorithm enabled us to measure even the smallest DDAF areas on the whole FAF image, even in cases with uneven illumination at the periphery, which has not been possible previously.

Analysis of the Outer Nuclear Layer Thickness

Spectral-domain OCT (SD-OCT) macular volume scans were performed using the Spectralis HRA+OCT device (Heidelberg Engineering, Heidelberg, Germany). These scans were obtained during the course of routine clinical care by a trained retinal imaging technician and included fovea-centered macular volume scan protocols that ranged from 20° to 30°, with 512 x 496 or 768 x 496 pixel resolution, comprised of 13, 19, 25, 37, 49 B-scans. The active eye-tracking system method (TruTrack™, Heidelberg Engineering, Heidelberg, Germany) enabled the acquisition of as much as

possible accurate OCT scan data, even in challenging patients with involuntary eye movement, poor attention and cooperation.

Outer nuclear layer (ONL) thickness provides an indirect measure of the photoreceptor cell loss and may be quantified on OCT scans. Segmentation of the ONL layer was done by a single grader (J.S.) semiautomatically, as the automated segmentation of retinal layers in pathologic changes of Stargardt disease (STGD1) led to many segmentation errors that required laborious manual correction. Therefore, first, an automatic segmentation of the ONL, extending from the outer plexiform layer (OPL) to the external limiting membrane (ELM), was obtained to the complete volume scan by using the in-built feature for automated segmentation in the Heidelberg Eye Explorer software (version 1.10.4.0). Then, OPL and ELM layers were reviewed, and manual correction was performed whenever necessary. A preserved ONL was considered only where OPL and ELM were distinguishable. If there was a particular area, where the two layers were not present, ONL was considered to be absent.

An ONL thickness map was created and analysed using the early treatment of diabetic retinopathy study (ETDRS) retinal grid. The ETDRS map was divided into 1, 3 and 6 mm rings, and the latter two further into quadrants (see Figure 1). The mean thickness of all nine zones was determined, expressed in microns (µm) and included in the final analysis. Additionally, ONL thickness of the outer ETDRS grid ring was analysed to evaluate ONL outside central DDAF (see Figure S7).

Electroretinography Testing

Electroretinography (ERG) testing was performed with an Espion visual electrophysiology testing system (Diagnosys LLC, Littleton, MA, USA). The recording electrode was an HK-loop placed in the fornix of the lower eyelid [2]. Large pattern ERG

(PERG) [3, 4] to the 30.7 x 23.6° checkerboard (Figure 1B) and full-field ERG (ffERG) [5] were recorded according to the standards of the International Society of Clinical Electrophysiology of Vision (ISCEV). PERG P50 amplitude was used to analyse the macular function. Rod system function was assessed with dark-adapted (DA) 0.01 ERG b-wave and DA 3.0 ERG a-wave amplitudes. Cone system function was tested with light-adapted (LA) 30 Hz ERG and LA 3.0 ERG b-wave amplitudes.

Microperimetry Examination

Retinal function was additionally analysed with microperimetry, a subjective, non-invasive visual field test. During retinal sensitivity assessment, fixation was continuously registered. Adjusted 10-2 Humphrey test grid was used, comprising 56 test loci, covering 20 x 20°. The test stimulus was white and was set to Goldmann size III. The stimulus intensity varied from 127 to 1.27 cd/m^2, corresponding to retinal sensitivities of 0 dB to 20 dB. A 4-2 threshold strategy was used. The fixation target was a red cross of a size the patient could perceive. Sensitivity values from all 56 test loci and fixation were then superimposed over a 55° FAF image using the MP1 microperimeter software (NAVIS software version 1.7.9, Nidek Technologies, Padova, Italy).

References

1. Strauss RW, Kong X, Ho A, et al. Progression of Stargardt disease as determined by fundus autofluorescence over a 12-month period: ProgStar report no. 11. *JAMA Ophthalmol.* 2019;137(10):1134-1145.

2. Hawlina M, Konec B. New noncorneal HK-loop electrode for clinical electroretinography. *Doc Ophthalmol.* 1992;81(2):253-259.

3. Lenassi E, Jarc-Vidmar M, Glavac D, Hawlina M. Pattern electroretinography of larger stimulus field size and spectral-domain optical coherence tomography in patients with Stargardt disease. *Br J Ophthalmol.* 2009;93(12):1600-1605.

4. Bach M, Brigell MG, Hawlina M, et al. ISCEV standard for clinical pattern electroretinography (PERG): 2012 update. *Doc Ophthalmol.* 2013;126(1):1-7.

5. Robson AG, Frishman LJ, Grigg J, et al. ISCEV standard for full-field clinical electroretinography (2022 update). *Doc Ophthalmol.* 2022;144(3):165-177.
