# Supplementary material for: ABCA4 Variant c.5714+5G>A in Trans With Null Alleles Results in Primary RPE Damage
Source: Invest Ophthalmol Vis Sci. 2023 Sep 20;64(12):33. doi: 10.1167/iovs.64.12.33 (PMC10516765; doi:10.1167/iovs.64.12.33)
Supplement: Supplement 3 [file iovs-64-12-33_s003.pdf]

TABLE S1. Primers for Quantitative Polymerase Chain Reaction

WT fragment	Primer orientation	Primer sequences (5'-3')	Fragment size (bp)
GUSB	Fwd	AGAGTGGTGCTGAGGATTGG	80
	Rev	CCCTCATGCTCTAGCGTGTC	
OCT3/4	Fwd	GTTCTTCATTCACTAAGGAAGG	101
	Rev	CAAGAGCATCATTGAACTTCAC	
ABCA4	Fwd	CATCCTGTTCCACCACCTCA	113
	Rev	CTGTGTCCTCCAACATGGCT	
PAX6	Fwd	GCTGCAAAGAAATAGAACATCC	111
	Rev	TTGGCTGCTAGTCTTTCTCG	
CRX	Fwd	CCCCAGTGTGGATCTGATG	116
	Rev	CAAACAGTGCCTCCAGCTC	
RCV	Fwd	ACACCAAGTTCTCGGAGGAG	108
	Rev	ACTTGGCGTAGATGCTCTGG	
OPN1SW	Fwd	TTCTTCTCCAAGAGTGCTTGC	97
	Rev	CCTTCCCACACACCATCTTC	
RPE65	Fwd	TTACTACGCTTGCACAGAGACC	105
	Rev	GCCCCATTGACAGAGACATAG	
OTX2	Fwd	TATCTTAAGCAACCGCCTTACG	75
	Rev	GGAGGGGTGCAGCAAGTC	
NRL	Fwd	GGAGGGGTGCAGCAAGTC	107
	Rev	AGCCAGTACAGCTCCTCCAG	
