# Supplementary material for: ABCA4 Variant c.5714+5G>A in Trans With Null Alleles Results in Primary RPE Damage
Source: Invest Ophthalmol Vis Sci. 2023 Sep 20;64(12):33. doi: 10.1167/iovs.64.12.33 (PMC10516765; doi:10.1167/iovs.64.12.33)
Supplement: Supplement 4 [file iovs-64-12-33_s004.pdf]

TABLE S2. Semi-quantitative analysis of splice products from photoreceptor precursor cells in the absence and presence of cycloheximid. Reverse transcription-polymerase chain reaction was performed in two biological replicates. On the Biological Replicate 2 (most representative), measurement of each fragment was performed in a technical duplicate (1 and 2), corrected for fragment size and expressed as area under the curve.

**Biological Replicate 1 (-CHX)**	Fragment	Raw intensity (replicate 1)	Raw intensity (replicate 2)	Fragment size	Correction factor	Corrected intensity (replicate 1)	Corrected intensity (replicate 2)	Product replicate 1	Product replicate 2	Average (%)
WT	WT	12061.309	11706.51	779	1.00000	12061.309	11706.51	1.0000	1.0000	100
c.5714+5G>A	WT	466.698	568.82	779	1.00000	466.698	568.82	0.7394	0.6450	**69.22**
	Δ 40	137.021	260.849	649	0.83312	164.4674253	313.0991849	0.2606	0.3550	**30.78**
	Δ 39/40	0	0	525	0.67394	0	0			
Total						631.1654253	881.9191849			

**Biological Replicate 1 (+CHX)**	Fragment	Raw intensity (replicate 1)	Raw intensity (replicate 2)	Fragment size	Correction factor	Corrected intensity (replicate 1)	Corrected intensity (replicate 2)	Product replicate 1	Product replicate 2	Average (%)
WT	WT	15370.409	14482.823	779	1.00000	15370.409	14482.823	1.0000	1.0000	100
c.5714+5G>A	WT	11490.56	10245.853	779	1.00000	11490.56	10245.853	0.8301	0.8092	**81.96**
	Δ 40	1742.477	1802.841	649	0.83312	2091.509373	2163.964775	0.1511	0.1709	**16.10**
	Δ 39/40	175.849	169.849	525	0.67394	260.926421	252.0235638	0.0188	0.0199	**1.94**
Total						13842.99579	12661.84134			

**Biological Replicate 2 (-CHX)**	Fragment	Raw intensity (replicate 1)	Raw intensity (replicate 2)	Fragment size	Correction factor	Corrected intensity (replicate 1)	Corrected intensity (replicate 1)	Product replicate 1	Product replicate 2	Average (%)
WT	WT	15809.35900	13736.995	779	1.00000	15809.359	13736.995	1.0000	1.0000	100
c.5714+5G>A	WT	10195.40900	8772.075	779	1.00000	10195.409	8772.075	0.8804	0.8700	**87.52**
	Δ 40	788.47700	774.648	649	0.83312	946.4153821	929.8163205	0.0817	0.0922	**8.70**
	Δ 39/40	295.26300	256.678	525	0.67394	438.1140514	380.861261	0.0378	0.0378	**3.78**
Total						11579.93843	10082.75258			

**Biological Replicate 1 (+CHX)**	Fragment	Raw intensity (replicate 1)	Raw intensity (replicate 2)	Fragment size	Correction factor	Corrected intensity (replicate 1)	Corrected intensity (replicate 1)	Product replicate 1	Product replicate 2	Average (%)
WT	WT	13609.40900	10345.631	779	1.00000	13609.409	10345.631	1.0000	1.0000	100
c.5714+5G>A	WT	11471.14600	10449.296	779	1.00000	11471.146	10449.296	0.7697	0.7543	**76.20**
	Δ 40	2528.03300	2485.841	649	0.83312	3034.418655	2983.775253	0.2036	0.2154	**20.95**
	Δ 39/40	268.67800	282.506	525	0.67394	398.6669752	419.1850933	0.0267	0.0303	**2.85**
Total						14904.23163	13852.25635			

- = absence; + = presence; CHX = cycloheximid; WT = wild-type; Δ = skipping.
Fragment quantities are expressed as percentage over the entire fragment mix.
