# Supplementary material for: ABCA4 Variant c.5714+5G>A in Trans With Null Alleles Results in Primary RPE Damage
Source: Invest Ophthalmol Vis Sci. 2023 Sep 20;64(12):33. doi: 10.1167/iovs.64.12.33 (PMC10516765; doi:10.1167/iovs.64.12.33)
Supplement: Supplement 5 [file iovs-64-12-33_s005.pdf]

TABLE S3. An Overview of All Genetic Variants in This Study Cohort

Patient ID	Sex	Allele 1 cDNA	Allele 1 protein	Allele 2 cDNA	Allele 1 protein
00785	M	c.5714+5G>A	c.[1622T>C;3113C>T]	p.[=,Glu1863Leufs*33]	p.[(Leu541Pro;Ala1038Val)]
00782	M	c.5714+5G>A	c.[1622T>C;3113C>T]	p.[=,Glu1863Leufs*33]	p.[(Leu541Pro;Ala1038Val)]
00809	F	c.5714+5G>A	c.[1622T>C;3113C>T]	p.[=,Glu1863Leufs*33]	p.[(Leu541Pro;Ala1038Val)]
00818	M	c.5714+5G>A	c.[1622T>C;3113C>T]	p.[=,Glu1863Leufs*33]	p.[(Leu541Pro;Ala1038Val)]
01139	M	c.[5461-10T>C;5603A>T]	c.5714+5G>A	p.[Thr1821Aspfs*6,Thr1821Valfs*13;(Asn1868Ile)]	p.[=,Glu1863Leufs*33]
00769	F	c.5175dup(;)5603A>T	c.5714+5G>A	p.(Thr1726Aspfs*61)(;)(Asn1868Ile)	p.[=,Glu1863Leufs*33]
00768	F	c.5175dup(;)5603A>T	c.5714+5G>A	p.(Thr1726Aspfs*61)(;)(Asn1868Ile)	p.[=,Glu1863Leufs*33]
00784	F	c.[1622T>C;3113C>T]	c.[1622T>C;3113C>T]	p.[(Leu541Pro;Ala1038Val)]	p.[(Leu541Pro;Ala1038Val)]
01138	F	c.[1622T>C;3113C>T]	c.[1622T>C;3113C>T]	p.[(Leu541Pro;Ala1038Val)]	p.[(Leu541Pro;Ala1038Val)]
00766	F	c.3259G>A	c.3259G>A	p.(Glu1087Lys)	p.(Glu1087Lys)
00773	F	c.6445C>T	c.6445C>T	p.(Arg2149*)	p.(Arg2149*)
00819	F	c.6445C>T	c.6445C>T	p.(Arg2149*)	p.(Arg2149*)
00799	F	c.6445C>T	c.6445C>T	p.(Arg2149*)	p.(Arg2149*)
00771	M	c.6445C>T	c.6445C>T	p.(Arg2149*)	p.(Arg2149*)
00787	M	c.5977del	c.5461-10T>C	p.(Ser1993fs)	p.[Thr1821Aspfs*6,Thr1821Valfs*13]
00783	F	c.2041C>T	c.4222delT	p.(Arg681*)	p.(Trp1408fs)
00793	F	c.5917del	p.(Val1973*)	c.5917del	p.(Val1973*)
00093	M	c.5977del	p.(Ser1993fs)	c.[1622T>C;3113C>T]	p.[(Leu541Pro;Ala1038Val)]
