# Supplementary material for: ABCA4 Variant c.5714+5G>A in Trans With Null Alleles Results in Primary RPE Damage
Source: Invest Ophthalmol Vis Sci. 2023 Sep 20;64(12):33. doi: 10.1167/iovs.64.12.33 (PMC10516765; doi:10.1167/iovs.64.12.33)
Supplement: Supplement 6 [file iovs-64-12-33_s006.pdf]

TABLE S4. Clinical Characteristics

Parameter	Group 1 patients (median, range)	Group 2 patients (median, range)	Mann-Whitney U test	Multiple linear regression			
				Genotype		Age at the exam	
			P value	B, 95% CI, β	P value	B, 95% CI, β	P value
Age at exam	34 years (16-63 years), N=7	22 years (12-65 years), N=11	P=0.930	N/A	N/A	N/A	N/A
Age of onset	17.0 years (11.0-35.0), N=7	8.0 years (6.0-10.0), N=11	**P<0.001**	N/A	N/A	N/A	N/A
Visual acuity	1.0 (0.5-2.5), N=7	1.8 (1.0-4.0), N=11	**P=0.044**	B=1.002, 95% CI [0.548, 1.457], β=0.514	**P<0.001**	B=0.046, 95% CI [0.032, 0.060], β=0.789	**P<0.001**
DDAF area	3.1 mm^2 (0.2-74.8), N=7	16.2 mm^2 (9.0-89.7), N=11	P=0.126	B=23.561, 95% CI [12.867, 34.255], β=0.355	**P<0.001**	B=1.827, 95% CI [0.029, 0.060], β=0.917	**P<0.001**
ONL thickness	13.9 μm (1.4-46.6), N=6	0.0 μm (0.0-0.0), N=11	**P<0.001**	B=−22.219, 95% CI [−32.483, −11.955], β=−0.758	**P<0.001**	B=−0.310, 95% CI [−0.607, −0.013], β=−0.366	**P=0.042**
PERG P50 amplitude	2.7 μV (0.0-4.9), N=7	0.0 μV (0.0-0.0), N=11	**P=0.001**	B=−2.842, 95% CI [−3.940, −1.743], β=−0.789	**P<0.001**	B−0.037, 95% CI [−0.070, −0.004], β=0.341	**P=0.031**
DA 0.01 ERG b-wave amplitude	116.2 μV (26.5-226.5), N=7	48.8 (0.0-112.5), N=11	**P=0.015**	B=−83.370, 95% CI [−118.190, −48.551], β=−0.647	**P<0.001**	B=−2.468, CI [−3.512, −1.424], β=−0.639	**P<0.001**
DA 3.0 ERG a-wave amplitude	86.6 μV (26.0-211.9), N=7	30.2 μV (0.0-65.2), N=11	**P=0.003**	B=−81.397, 95% CI [−115.196, −47.598], β=−0.699	**P<0.001**	B−1.898, 95% CI [−2.911, −0.885], β=−0.544	**P=0.001**
LA 30 Hz ERG amplitude	41.8 μV (8.5-81.6), N=7	0.0 μV (0.0-36.0), N=11	**P=0.004**	B=−39.190, 95% CI [−54.714, −23.666], β=−0.739	**P<0.001**	B=−0.763, 95% CI [−1.228, −0.297], β=−0.480	**P=0.003**
LA 3.0 ERG b-wave amplitude	53.2 μV (4.2-116.5), N=7	0.0 μV (0.0-38.2), N=11	**P=0.003**	B=−50.142, 95% CI [−71.351, −28.933], β=−0.731	**P<0.001**	B=−0.935, 95% CI [−1.571, −0.299], β=−0.454	**P=0.007**

ONL = outer nuclear layer; DDAF = definitely decreased autofluorescence; PERG = pattern electroretinography; DA = dark adapted; LA = light adapted; N/A = not applicable; N = number of patients; B = unstandardized regression coefficient; brackets denote 95% confidence intervals; β = standardized regression coefficient. The values in bold indicate statistical significance defined as P value < 0.05. Numerical values are expressed with median values and range.
