# Supplementary material for: ABCA4 Variant c.5714+5G>A in Trans With Null Alleles Results in Primary RPE Damage
Source: Invest Ophthalmol Vis Sci. 2023 Sep 20;64(12):33. doi: 10.1167/iovs.64.12.33 (PMC10516765; doi:10.1167/iovs.64.12.33)
Supplement: Supplement 7 [file iovs-64-12-33_s007.pdf]

TABLE S5. Effect of Age on PERG P50 Amplitude, ONL Thickness and DDAF Area

Parameter	Group 1		Group 2	
	Simple linear regression		Simple linear regression	
	ANOVA results	B and β Coefficients	ANOVA results	B and β Coefficients
PERG P50 amplitude	R^2=0.780, $R^2_{adjusted}$=0.736, $F(1, 5)$=17.689, **P=0.008**	B=−0.105, 95% CI [−0.168, −0.041], β=−0.883	R^2=0.929, $R^2_{adjusted}$=0.921, $F(1, 9)$=117.470, **P<0.001**	B=1.961, 95% CI [1.551, 2.370], β=0.964
ONL thickness	R^2=0.807, $R^2_{adjusted}$=0.758, $F(1, 4)$=16.695, **P=0.015**	B=−0.947, 95% CI [−1.591, −0.304], β=−0.898	N/A	N/A
DDAF area	R^2=0.884, $R^2_{adjusted}$=0.861, $F(1, 5)$=38.043, **P=0.002**	B=1.582, 95% CI [0.923, 2.241], β=0.940	N/A	N/A

PERG = pattern electroretinography; ONL = outer nuclear layer; DDAF = definitely decreased autofluorescence; N/A = not applicable; B=unstandardized regression coefficient; brackets denote 95% confidence intervals; β=standardized regression coefficient. The values in bold indicate statistical significance defined as P value < 0.05.
